# Divergence in Transcriptional and Regulatory Responses to Mating in Male and Female Fruitflies

**DOI:** 10.1038/s41598-019-51141-9

**Published:** 2019-11-06

**Authors:** Emily K. Fowler, Thomas Bradley, Simon Moxon, Tracey Chapman

**Affiliations:** 1 School of Biological Sciences, University of East Anglia, Norwich Research Park, Norwich, NR4 7TJ UK; 2 Earlham Institute, Norwich Research Park, Norwich, NR4 7UZ UK

**Keywords:** Transcriptomics, Sexual selection

## Abstract

Mating induces extensive physiological, biochemical and behavioural changes in female animals of many taxa. In contrast, the overall phenotypic and transcriptomic consequences of mating for males, hence how they might differ from those of females, are poorly described. Post mating responses in each sex are rapidly initiated, predicting the existence of regulatory mechanisms in addition to transcriptional responses involving *de novo* gene expression. That post mating responses appear different for each sex also predicts that the genome-wide signatures of mating should show evidence of sex-specific specialisation. In this study, we used high resolution RNA sequencing to provide the first direct comparisons of the transcriptomic responses of male and female *Drosophila* to mating, and the first comparison of mating-responsive miRNAs in both sexes in any species. As predicted, the results revealed the existence of sex- and body part-specific mRNA and miRNA expression profiles. More genes were differentially expressed in the female head-thorax than the abdomen following mating, whereas the opposite was true in males. Indeed, the transcriptional profile of male head-thorax tissue was largely unaffected by mating, and no differentially expressed genes were detected at the most stringent significance threshold. A subset of ribosomal genes in females were differentially expressed in both body parts, but in opposite directions, consistent with the existence of body part-specific resource allocation switching. Novel, mating-responsive miRNAs in each sex were also identified, and a miRNA-mRNA interactions analysis revealed putative targets among mating-responsive genes. We show that the structure of genome-wide responses by each sex to mating is strongly divergent, and provide new insights into how shared genomes can achieve characteristic distinctiveness.

## Introduction

Mating is well-known to induce extensive behavioural and physiological changes in animals of many taxa. These include changes to fecundity, longevity, immunity, chemical signalling and sexual receptivity^1–4^. Post mating responses (PMRs) can be initiated within seconds or minutes or may build up over several hours or days^5,6^. They also vary in duration and may be sustained either temporarily, or permanently throughout life^7^. PMRs may act to optimise physiological and behavioural processes in mated individuals to facilitate subsequent reproductive effort or behaviour. However, the form of PMRs is expected to diverge significantly between the sexes. For example, in species in which both sexes mate multiply, mated females often show refractory responses associated with lowered willingness to mate and their removal from the mating arena in order to support and sustain the production of fertile eggs, at least until sperm supplies become depleted and sexual receptivity returns. In contrast, mated males are likely to be subject to selection pressures to minimise any refractory period, replenish ejaculates and rapidly re-enter reproductive competition in the mating arena.

PMRs are particularly well-studied in females of *Drosophila melanogaster* (reviewed in^8,9^). During copulation, a male *D. melanogaster* transfers a suite of well over 150 seminal fluid proteins (Sfps) along with thousands of sperm to the female^10,11^. Many of the changes which occur in females following mating are induced by Sfps, and the magnitude of female PMRs appears to be dependent upon the quantity and relative composition of Sfps received in the ejaculate^12,13^. Sfp receipt increases oogenesis, ovulation and feeding^14^, reduces siesta sleep^15^, increases female aggression towards other females^16^ and reduces sexual receptivity toward males^17^. Other physiological effects of Sfps include facilitation of sperm storage and retention^18,19^, changes to immune gene expression^20,21^ and to nutrient and water balance^22,23^. The adaptive modulation of PMRs appears to be facilitated by the highly precise^24^ and socially-flexible^25,26^ expression of Sfp-encoding genes.

In contrast, in males surprisingly little is known about the physiological or behavioural changes associated with mating. Some type of refractory period is generally noted, as following mating, both sperm and Sfps must be replenished. Data are scarce, but it is suggested that in general seminal fluids are in more limited supply than are sperm themselves^27–30^. The refuelling of a full complement of Sfps in particular is known in some cases to take time, e.g. over 24 h in *D. melanogaster* males^29–31^. Potentially associated with this, physiological changes to the ejaculatory duct have also been noted in mated *D. melanogaster* males^32,33^. Aside from these responses, there is some evidence from targeted gene expression studies that males invest in immune response molecules following mating, similarly to females^34^. In contrast, while females increase and adapt their nutritional intake following mating, the same is not true for males^35^.

Since males and females share the vast majority of their genome, sexually dimorphic traits such as PMRs are predicted to arise from the sex-specific regulation of gene expression^36^. Hence measurements of genome-wide, mating-responsive gene expression profiles can provide insight into the underlying mechanisms involved in PMRs in both sexes. Transcriptomic profiles of PMRs have been generated for females of multiple insect species, e.g. the Mediterranean fruitfly *Ceratitis capitata*^37^; the honey bee *Apis mellifera*^38^; mosquitoes *Anopheles gambiae*^39^ and *Aedes aegypti*^40^ and the seed beetle *Callosobruchus maculatus*^41^. In transcriptomic studies of PMRs in female *D. melanogaste*r, several have focussed on profiling responses in the whole female fly^42–47^. Others have profiled individual body parts, such as the female reproductive tract^48–50^, or heads^51^ which has helped to reveal additional complexity which can sometimes be obscured by whole body arrays and profiles^52^. Several other studies have also profiled the responses of females to the receipt of sperm or Sfps^44,45,53,54^. Collectively, this work has revealed that PMRs can induce pervasive, genome-wide gene expression changes in reproductive, sensory and immune system genes with some similarities between signatures of mating and aging processes^46^.

In contrast to females, transcriptomic studies of PMRs in males are scarce. Gene expression profiles of male and female *C. capitata* and *C. maculatus* revealed the presence of distinct, sex-specific transcriptional responses to mating^37,41^. However, for *D. melanogaster*, no direct comparison of the transcriptomic mating response by males and females has previously been undertaken, and data on male responses are restricted to a single study using head tissue^55^.

The timing of the different facets of PMRs in both sexes is also highly variable and distinct. This suggests that mechanisms in addition to expression changes in coding genes are likely to be important contributors to PMRs and should themselves show sex-specificity. Some PMRs are extremely rapid and may rely upon the release of neurotransmitters^56^ or the actions of regulatory molecules. We have scant data so far of these aspects of PMRs, particularly in how changes in gene regulation versus gene expression are linked. Hence a significant part of our mechanistic understanding of the responses of both sexes to mating is still missing. Consistent with the idea that PMRs are achieved by a range of qualitatively different responses, recent data in female *D. melanogaster* show that regulatory molecules, such as miRNAs, can also change in response to mating^46,57^. miRNAs are a group of small non-coding RNA molecules which play a key role in post-transcriptional gene regulation by binding complementary mRNA transcripts, inhibiting their translation into peptides. Well-known for their role in gene regulation during development, miRNAs are increasingly implicated in the expression of adult phenotypes, including the regulation of Sfps^24^, male and female fertility and ovary morphology^58^. These recent findings predict significant changes to the expression of coding and regulatory non-coding genes following mating in both sexes, but as yet there has been no genome-wide analysis of non-coding RNA responses to mating in males. Study of the expression profiles of miRNAs in tandem with mRNAs also offer the potential for new insights into the regulatory processes underlying the changes in transcript abundance.

In this study, we addressed the omissions noted above by testing two predictions: (i) that there are significant changes to the expression of both coding and regulatory non-coding genes between virgin and mated flies in both sexes, and (ii) that the mode and nature of PMR gene expression profiles of each sex are markedly different. The data supported both predictions. For the female head-thorax (HT) and male abdomen (Ab) tissues, >2000 genes were differentially expressed (DE) between virgin and mated status. Interestingly, for the female HT the majority of DE genes were downregulated following mating, while many of the same genes were upregulated in the mated male Ab. In contrast, only 125 genes were DE after mating in the female Ab, while mating did not significantly impact gene expression in the male HT. The magnitude of genome-wide change showed sex specificity and was much greater in females, with ~50% vs 15% of DE genes showing greater than two-fold change in females vs males, respectively. We identified novel mating-induced miRNAs in the abdomens of both sexes, with changes occurring in fewer miRNAs in males than in females.

## Results

### Sequencing QC

**Figure 1:**
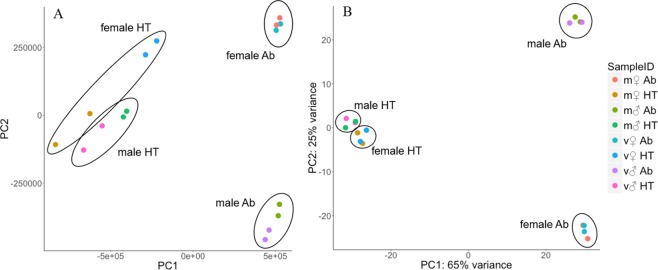
Principle component analyses of mRNA-sequencing (**A**) and miRNA sequencing data (**B**). Points are coloured by sample type: mated (m), virgin (v), male (♂), female (♀), abdomen tissue (Ab) or head-thorax tissue (HT).

To determine the transcriptomic profiles of mated and virgin flies, we conducted high-throughput RNA sequencing (RNA-seq). We extracted the mRNA and small RNA (sRNA) fractions from a total of 16 samples, consisting of two treatments (virgin vs. three hours post-mated), two sexes, two body-parts (HT and Ab) and two biological replicates. FASTQ files generated from the sequencing reads were checked using FastQC (Babraham Bioinformatics) and no significant quality issues were discovered. RNA-seq reads had an average pseudo-alignment rate of 77.61% to the transcriptome (min 64.63%, max 85.1%, Supplementary Table S1a), and sRNA reads had an average of 84.97% alignment rate to the genome (min 82.49%, max 88.63%, Supplementary Table S1b). PCA plots showed clustering by sample for both mRNA-seq and sRNA-seq datasets (Fig. 1). Variation between replicates was generally very low. During differential expression analysis using the DESeq2 package^59^, coding genes or miRNA exhibiting high variability in expression values between replicates are penalised, so genes are only called DE if the variation between treatments is over and above that attributable to the replicates. Size distribution profiles of sRNA read length showed peaks at 22 and 23 nt, corresponding to the expected length for miRNAs (Supplementary Table S1c). In previous *Drosophila* sequencing studies, we and others^46,60^ have noted a read length peak at 30 nt, accounting for >90% of the total reads. This fraction may contain some longer sRNA species such as piRNAs, but is dominated by reads corresponding to the highly abundant 30 nt 2S rRNA fraction found in insect species. To remove this fraction, we incorporated a complimentary “blocking oligo” into the library construction^60^, which successfully prevented adaptor ligation of 2S rRNA, thus increasing the proportion of reads derived from miRNAs.

### Mating-Responsive mRNAs

The total numbers of genes showing DE in response to mating varied widely across sex and tissue type (Table 1; Supplementary Table S2a–d), as did the magnitude of the fold change in expression. The female HT and male Ab tissues had the greatest number of mating-responsive genes, with over 2000 in each case. In contrast, the number of genes affected by mating was much lower in the female Ab, in which only 125 genes were DE between the virgin and mated treatments. Strikingly, no genes were DE between the HT of virgin versus mated males with a q-value of < 0.05. Replicate to replicate variability of virgin and mated male head-thorax samples was comparable with other samples (Supplementary Fig. S1). Therefore, the absence of statistically significantly differentially expressed genes cannot be explained by replicate variability and appears to be a biologically relevant effect. The magnitude of change in gene expression was greater in females than males, with ~50% of DE genes showing greater than two-fold change regardless of body-part. Gene expression changes in the male abdomen, though involving numerous genes, were more subtle, with only 15% of DE genes showing over two-fold change. To detect signatures of enriched function amongst the DE genes we performed gene ontology (GO) enrichment analyses on all sets of mating responsive genes (Table 2, Supplementary Table S3a–h). We also carried out GO analyses on the subset of genes in each sex/tissue type which exceeded a fold-change threshold of two (Supplementary Table S4a–f).

**Table 1:** Total numbers of mating-responsive mRNAs and miRNAs in male (♂) and female (♀) head-thorax and abdomen tissues. Numbers of significant DE genes with a fold-change difference of >2 are shown in parentheses.

	Significance threshold	Total DE genes	Upregulated in mated flies (>2 fold)	Downregulated in mated flies (>2 fold)
**mRNA**				
♀ abdomen	*p* < 0.05	628	475 (**110**)	153 (**32**)
	*FDR q* < 0.05	125	106 (**50**)	19 (**12**)
♀ head-thorax	*p* < 0.05	3372	1206 (**224**)	2166 (**1036**)
	*FDR q* < 0.05	2040	628 (**144**)	1412 (**804**)
♂ abdomen	*p* < 0.05	3607	2273 (**355**)	1334 (**38**)
	*FDR q* < 0.05	2068	1507 (**296**)	561 (**19**)
♂ head-thorax	*p* < 0.05	329	265 (**19**)	64 (**2**)
	*FDR q* < 0.05	0	0	0
**miRNA**				
♀ abdomen	*p* < 0.05	16	12	4
	*FDR q* < 0.05	4	3	1
♀ head-thorax	*p* < 0.05	10	4	6
	*FDR q* < 0.05	0	0	0
♂ abdomen	*p* < 0.05	9	2	7
	*FDR q* < 0.05	2	0	2
♂ head-thorax	*p* < 0.05	2	2	0
	*FDR q* < 0.05	0	0	0

**Table 2:** Representative GO biological process terms significantly enriched among the mating-responsive genes in the female abdomen (♀ Ab), male head-thorax (♂ HT) and male abdomen (♂ Ab). Term enrichment analysis was performed using GOrilla.

Biological process term	♀ Ab (up) gene count	♀ HT (down) gene count	♂ Ab (up) gene count
electron transport chain	6	44	35
translation	17	79	91
organic substance biosynthetic process	25	227	231
organonitrogen compound metabolic process	45	395	436
primary metabolic process	56	543	589
organic substance metabolic process	60	599	643
metabolic process	67	694	720
mitochondrial protein processing		5	5
cell redox homeostasis		16	19
generation of precursor metabolites and energy		70	44
cofactor metabolic process		58	48
organophosphate metabolic process		73	63
oxidation-reduction process		136	93
cellular amide metabolic process		94	120
macromolecule biosynthetic process		117	125
organonitrogen compound biosynthetic process		153	148
small molecule metabolic process		168	149
cellular nitrogen compound biosynthetic process		154	160
cellular biosynthetic process		222	223
biosynthetic process		230	234
cellular nitrogen compound metabolic process		260	293
cellular macromolecule metabolic process		281	316
nitrogen compound metabolic process		481	535
cellular metabolic process		572	613
cellular process		843	899
cellular response to unfolded protein			8
translational initiation			15
cellular component biogenesis			16
Golgi organization			28
rRNA metabolic process			30
Golgi vesicle transport			31
protein folding			48
protein-containing complex subunit organization			90
macromolecule localization			109
organic substance transport			123
establishment of localization in cell			124
proteolysis			129
cellular localization			132
catabolic process			136
cellular protein metabolic process			226
protein metabolic process			329
macromolecule metabolic process			453
regulation of autophagy of mitochondrion		5	
muscle system process		9	
reactive oxygen species metabolic process		11	
cellular aldehyde metabolic process		12	
mitochondrial transport		22	
antibiotic metabolic process		25	
monovalent inorganic cation transport		29	
cellular homeostasis		43	
carbohydrate metabolic process		56	
carbohydrate derivative metabolic process		80	
drug metabolic process		81	
rRNA 3′-end processing	3		
response to heat	7		
multi-organism process	13		

#### DE in the female abdomen

125 protein coding genes were responsive to mating in female abdomens. Most of these (106) were upregulated in mated females. A GO enrichment analysis of all upregulated genes revealed a significant over-representation (FDR q-value < 0.05) of 30 biological process terms (Supplementary Table S3a). Many of the terms were related to translation and peptide synthesis due to the presence of 17 ribosomal protein encoding genes. More generally, “protein metabolism” was enriched which, aside from ribosomal protein genes, involved 16 peptidase-encoding genes, including the spermathecal endopeptidases Send1 and Send2. At least six terms were related to “multi-organism process” and two terms involving response to heat were also enriched, generated by the presence of 13 genes encoding immune system, or stress response proteins. Additionally, the term “electron transport chain” was enriched, associated with six genes – *blw*, *CG3835*, *CG3731*, *CG4169*, *ND75* and *RFeSP*. Of the 106 significantly upregulated genes, 50 exceeded the two fold-change threshold. A GO analysis of these revealed four significant biological process terms, three of which were related to “response to bacterium”, and involved eight different genes. The other enriched term, “proteolysis”, consisted of 12 genes (Supplementary Table S4a). Of the 19 genes which were downregulated in mated female abdomens, there were no terms with an FDR q-value < 0.05. However, five genes related to “carbohydrate metabolic process” were present in this subset: *CG32444*, *Mal-A1*, *Mal-A7* and *Mal-A8*, and *tobi* (Supplementary Table S3b).

#### DE in the female head-thorax

2040 genes showed DE between virgin and mated females in the HT. Of these, 628 had higher expression in mated females. An enrichment analysis of the upregulated genes did not return any terms with an FDR q-value < 0.05, although terms associated with ncRNA processing fell just below the significance threshold (Supplementary Table S3c). Excluding low fold-change (<2 FC) DE genes from the GO analysis highlighted enrichment in two terms related to rRNA processing (Supplementary Table S4b). Of the 1412 downregulated genes, 141 biological process terms were significantly over-represented (Supplementary Table S3d). Almost all were linked to metabolic processes involving the generation of precursor metabolites and energy and the oxidation-reduction process, nucleoside phosphate metabolic process, and organonitrogen compound metabolic process. There were also terms associated with carbohydrate metabolism and oxoacid metabolism. Specific terms which were significantly enriched included “translation”, “ATP biosynthetic process”, “glycolytic process”, “muscle contraction” and “drug metabolic process”. More than half of the downregulated HT genes exceeded the two fold-change threshold. A GO analysis on this subset returned 106 enriched biological process terms, again mostly related to organonitrogen compound biosynthesis and energy metabolism, as well as carbohydrate metabolism and many other metabolic processes (Supplementary Table S4c).

#### DE in the male abdomen

2068 protein-coding genes were DE between virgin and mated male abdomens, and 1507 of these were upregulated in the mated flies. A GO enrichment analysis of the upregulated genes returned 97 biological process terms with an FDR q-value < 0.05 (Supplementary Table S3e). At least 35 of the terms were related to the transport and localization of organic substances and proteins, and protein folding. Another two terms were related to proteolysis. The remaining terms were connected to metabolic processes. Similarly to the downregulated genes in the female HT, these included terms related to translation and energy generation. Additionally, the term “translational initiation” was enriched, driven by the presence of genes encoding eukaryotic translation initiation factor. Only 20% of the upregulated male abdomen genes had a fold change of over two, but this subset was enriched for 32 terms, mostly generated by the presence of ~50 ribosomal protein genes (Supplementary Table S4d). These terms included “translation” and “organonitrogen compound biosynthesis”. Terms involving energy generation also remained overrepresented. Among the higher fold-change subset, “defence response to Gram-positive bacterium” was enriched, whereas the terms connected to protein transport and folding were absent. The 561 downregulated genes were not significantly enriched for any biological process terms, although the term “response to nutrient levels” fell just below the significance threshold (Supplementary Table S3f). Only 19 genes were over two-fold DE in the downregulated set. A GO analysis of this subset also did not return any significantly enriched terms. However, six terms had a p-value of > 0.05 and were all connected to glutamate receptor signalling, involving four genes – *Rdl*, *Syt1*, *CG32447* and *mtt* (Supplementary Table S4e).

#### DE in the male head-thorax

Strikingly, no mating-responsive genes met the stringent q-value < 0.05 threshold in the comparison of virgin and mated male head-thorax tissue. However, a GO enrichment analysis on 329 DE genes with a p-value < 0.05 showed that, similarly to the male abdomen, terms involving ribosomal protein genes, such as “translation” were over-represented in the 264 upregulated genes (Supplementary Table S3g). When we analysed the 19 upregulated genes with greater than two-fold DE, 11 terms associated with defence response were enriched, represented by five genes – *AttB*, *CecA2*, *LysX*, *CecC* and *Drsl2* (Supplementary Table S4f). GO analysis of the 64 downregulated genes returned terms involving molybdopterin cofactor processing, caused by three genes – *cin*, *Mocs2* and *CG42503* (Supplementary Table S3h). All but two of the downregulated genes had a fold change of over two, so no further GO analyses were carried out on these genes.

#### Comparison of sex- and tissue-specific profiles of mRNAs

**Figure 2:**
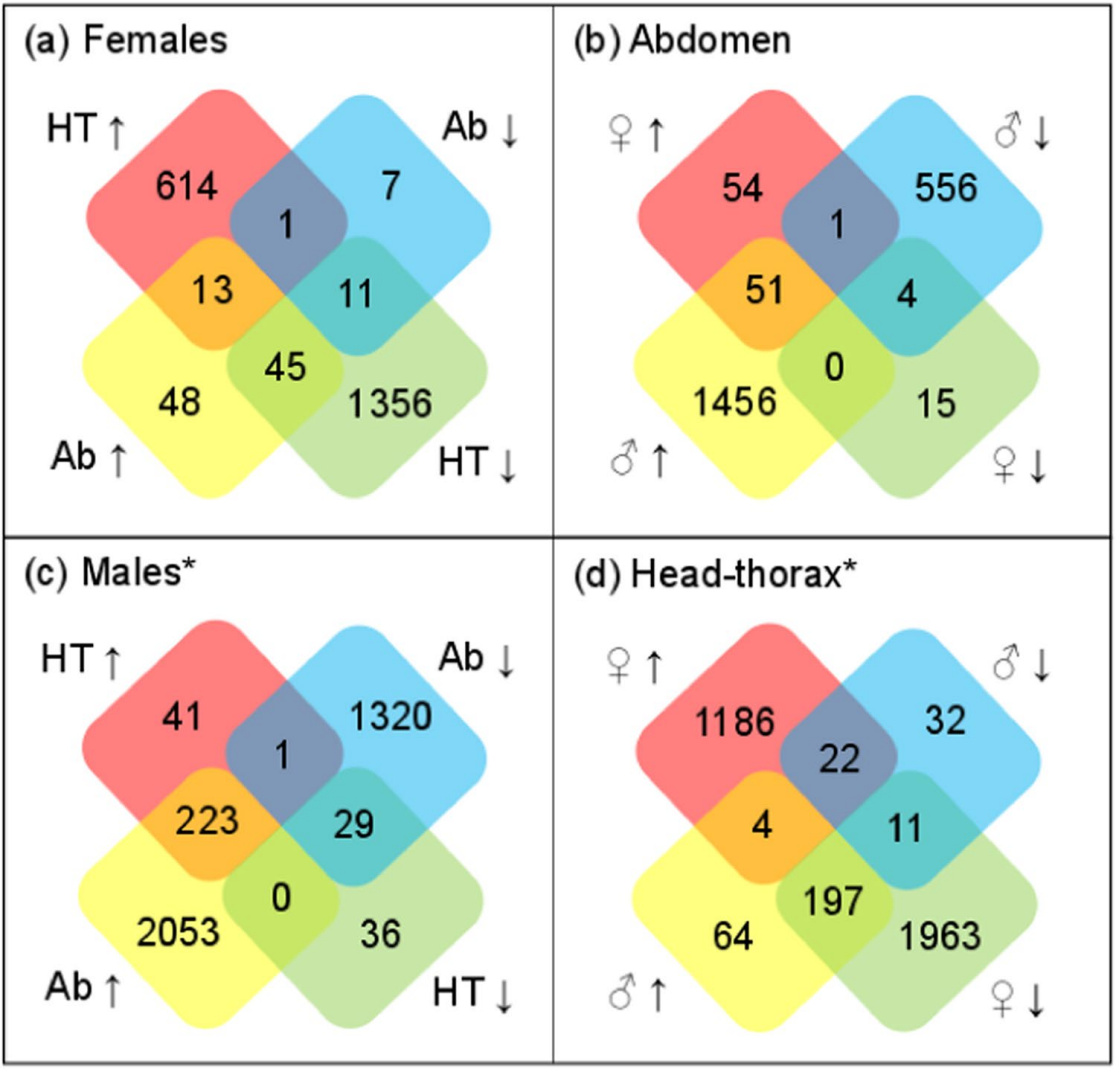
Overlap of the identities of up- and downregulated genes in response to mating in the different transcriptomes. (**a**) Numbers of DE genes upregulated (↑), or downregulated (↓) in response to mating in female abdomen (Ab) or head-thorax (HT). (**b**) Numbers of DE genes up- or downregulated in female or male Ab tissues. (**c**) Numbers of DE genes upregulated, or downregulated in response to mating in male Ab or HT (**d**) Numbers of DE genes upregulated, or downregulated in response to mating in female (♀) or male (♂) HT tissue. *DE calling based on a significance threshold of p < 0.05.

We compared the mating responsive genes in each sex and tissue type to one another, to examine differences in the transcriptomic profiles between males and females and between different body parts (Fig. 2). For comparisons of female HT and abdomen tissues, and female and male abdomens, we were able to use the set of DE genes with an FDR q-value of < 0.05 (Table 1). However, since there were no such genes falling below that cut-off in the male HT, we produced extended DE gene lists with a p-value of < 0.05 for the male HT, Ab and the female HT. This less stringent threshold for DE calling allowed us to explore the most significant changes in gene expression in the mated male HT, and make fair comparisons with the male Ab and female HT. GO analyses were conducted on the overlapping genes (Supplementary Table S5) to detect shared signatures of functional enrichment.

We first compared the DE genes in the female HT and female Ab, and found a total overlap of 70 genes (Fig. 2a). Of these, 13 genes were upregulated in response to mating in both the Ab and HT tissues, including the turandot protein genes *TotA*, *TotC* and *TotX*, and the gene encoding juvenile hormone esterase (Jhe) (Supplementary Table S5a). There were also 11 genes downregulated in both tissues in females, including the five genes predicted to be involved in carbohydrate metabolism, mentioned earlier (Supplementary Table S5b). Another 46 genes were differentially expressed in both body-parts, but in opposing directions. All but one of the opposing genes were downregulated in response to mating in the HT, and upregulated in the Ab. Over-represented among these were 14 ribosomal protein genes, and six genes encoding proteins involved in oxidative phosphorylation (*ND-75*, *RFeSP*, *UQCR-C1*, *UQCR-C2*, *COX8*, *blw*) (Supplementary Table S5c).

We next considered whether the mating responsive genes of the female Ab were also DE in the male Ab (Fig. 2b). In this case almost all overlapping genes were expressed in the same direction, i.e. 51 genes were upregulated in both the female and male Ab in response to mating. Again, a GO analysis on the 51 genes revealed an enrichment in the terms “translation”, and “electron transport chain” (Supplementary Table S5d). Using the less stringent DE calling, we next compared all DE genes with a p-value < 0.05 in the male HT, to those from the male Ab (Fig. 2c). In contrast to the female, overlapping DE genes in the male tissues were always DE in the same direction (with the exception of one gene), i.e. 223 genes were upregulated in both HT and Ab, and 29 genes were downregulated in both tissues. Once again, GO terms associated with translation were enriched amongst the 223 overlapping upregulated genes, while there were no significantly enriched terms for the downregulated genes (Supplementary Table S5e,f).

Finally, we compared the male and female HT body parts (Fig. 2d). Unlike in the Ab samples, where overlapping DE genes tended to be expressed in the same direction in both sexes, in the HT they were more likely to be expressed in opposite directions. For example, we found 197 genes that were upregulated in the male HT, but downregulated in the female HT. GO analysis of the overlapping genes again revealed an enrichment of terms related to translation, and organonitrogen compound metabolism (Supplementary Table S5g).

#### Comparison of mRNA profiles with existing studies

Several previous studies have investigated transcriptomic changes in response to mating in *D. melanogaster* females, with variation in the tissue type analysed, and the time-point captured following mating (Supplementary Table S6a). To compare the mating-responsive genes found in previous studies with our data here, we selected the studies most closely matching our experimental protocol. For example, we compared our female Ab samples to those studies that used either whole females, or reproductive tracts, and our female HT samples to those using heads or whole females. A summary of the studies and the numbers of mating-responsive genes identified in each one can be found in Supplementary Tables S6a,b.

First we examined the number of mating-responsive genes in our dataset which were also DE in other studies, regardless of the direction in expression change (Supplementary Table S6b–f). For our female abdomen samples, 65% of DE genes were also found to be mating-responsive in at least one other study. For the HT samples there was less, but still considerable, agreement with 29% of our DE genes also responding to mating in at least one other study. When we also considered the direction of DE, for the female Ab samples, 34.9% of our upregulated genes were also upregulated in at least one other study, and 63.2% of downregulated genes were downregulated in other studies. For the HT, 19.4% of genes increasing expression following mating in our study also did so in at least one previous study. For the downregulated genes in the head-thorax, agreement with other studies fell to 8.9% when direction of expression was taken into account. Interestingly, the female HT genes which showed opposing direction of DE in our study compared to previous studies were enriched for genes involved in translation, which we have shown to exhibit opposing expression in the head-thorax in comparison to abdomen samples. Studies measuring transcriptomic responses in whole flies would not have captured these body-part specific patterns.

To identify individual genes consistently found to be mating-responsive throughout the literature, we examined in more detail the DE genes found in our study and at least three previous studies. For upregulated genes in the abdomen, four genes were found to increase expression following mating in three other studies (*Uro*, *jhamt*, *CG17234*, *CG3290*), two were found in four other studies (*su(r)* and *CG17239*) and another two in five other studies (*CG31324* and *Send2*). For upregulated genes in the head-thorax, *CG6910*, *CG3036* and *Uro* were found in three other studies, *CG31324* was found in four other studies and *fit* was found in five other studies.

### Sex- and tissue-specific miRNA profiles

Reads from the sRNA-Seq output aligned to 401 mature miRNAs. The principle component analysis revealed that the miRNA profiles of HT and Ab tissues were distinct (Fig. 1). Furthermore, the miRNA profile of male and female Ab body parts differed significantly. This was in contrast to the HT, in which both sexes had similar profiles (Fig. 1). To investigate the sex- or tissue-bias in miRNA expression, we conducted a differential expression analysis between sexes and tissues (Supplementary Table S7a–d). A comparison of HT and Ab body-parts revealed that 83 miRNAs and 71 miRNAs were HT or Ab biased, respectively, in both sexes (Table 3). For the Ab-biased miRNAs, 45 were biased in males only, and 29 in females. There were fewer HT-biased miRNAs which were specific to one sex - 21 miRNAs were HT-biased in males only, and 14 miRNAs were HT-biased in females only. When males were compared to females, as expected from the PCA, most sex-biased miRNAs were specific to the Ab (97 male-biased, 42 female biased), with fewer miRNAs specific to the head-thorax (9 male-biased, 12 female-biased). There were also some miRNAs that were male- or female-biased in both body parts (8 male-biased, 12 female-biased) (Table 4). We examined the identities of the sex- and tissue-biased miRNAs and found strong concordance with a meta-analysis on sex-bias using publicly available sRNA-Seq data^61^. Of the 37 female-biased miRNAs identified in the meta-analysis, 31 were also female-biased in this study. Similarly, 26 of the 28 male-biased miRNAs identified were also male-biased here. The fact that the majority of sex-biased miRNAs were also abdomen-biased in this study is consistent with the fact that male- or female-biased miRNAs tend to be expressed in the testes or ovaries, respectively^61^.

**Table 3:** miRNAs with tissue-biased expression in both sexes, or in males or females only. Numbers in paratheses are the total miRNAs in each category, inclusive of 3p and 5p strands where both are differentially expressed.

Head-thorax biased miRNAs			Abdomen biased miRNAs		
*Both sexes (83)*			*Both sexes (71)*		
bantam-3p, 5p	miR-193-3p, 5p	miR-4952-5p	miR-1007-5p	miR-306-5p	miR-960-3p, 5p
let-7-5p	miR-210-3p, 5p	miR-4960-3p	miR-1012-3p	miR-308-3p, 5p	miR-961-3p, 5p
miR-1000-3p, 5p	miR-219-5p	miR-4968-5p	miR-1015-3p	miR-310-3p	miR-962-3p, 5p
miR-1001-3p, 5p	miR-2489-3p	miR-7-5p	miR-10-3p, 5p	miR-311-3p, 5p	miR-963-3p, 5p
miR-1004-3p, 5p	miR-252-3p, 5p	miR-87-3p, 5p	miR-12-3p, 5p	miR-312-3p, 5p	miR-964-3p, 5p
miR-1005-3p	miR-263b-5p	miR-927-5p	miR-184-5p	miR-313-3p, 5p	miR-982-5p
miR-1006-3p	miR-276a-3p, 5p	miR-929-3p	miR-2494-3p, 5p	miR-314-3p, 5p	miR-983-3p, 5p
miR-1009-3p	miR-276b-3p, 5p	miR-932-3p, 5p	miR-263a-3p, 5p	miR-316-3p, 5p	miR-984-3p, 5p
miR-1017-3p	miR-277-3p, 5p	miR-9383-3p	miR-275-3p	miR-31a-5p	miR-991-3p
miR-11-3p	miR-278-3p	miR-957-3p, 5p	miR-279-3p, 5p	miR-31b-5p	miR-997-5p
miR-124-3p, 5p	miR-284-3p, 5p	miR-969-3p	miR-281-1-5p	miR-33-5p	miR-9b-3p, 5p
miR-125-5p	miR-285-3p, 5p	miR-970-3p, 5p	miR-281-2-5p	miR-4919-3p, 5p	miR-9c-5p
miR-133-3p, 5p	miR-2c-3p, 5p	miR-971-3p, 5p	miR-281-3p	miR-92a-3p	miR-iab-4-3p
miR-137-5p	miR-307a-3p	miR-981-3p, 5p	miR-282-5p	miR-956-3p, 5p	miR-iab-8-5p
miR-13a-3p, 5p	miR-315-3p, 5p	miR-987-5p	miR-283-3p, 5p	miR-958-3p, 5p	
miR-13b-1-5p	miR-317-5p	miR-990-5p	miR-304-3p, 5p	miR-959-3p, 5p	
miR-1-3p, 5p	miR-34-3p, 5p	miR-993-3p, 5p	***Females only (29)***		
miR-14-3p	miR-4945-5p	miR-998-5p	miR-1008-5p	miR-2a-3p	miR-92b-3p
miR-190-5p	miR-4951-5p	miR-999-3p	miR-1014-5p	miR-2b-2-5p	miR-9372-5p
***Females only (14)***			miR-11-5p	miR-2b-3p	miR-989-3p, 5p
miR-100-3p	miR-307a-5p	miR-4956-5p	miR-13b-2-3p	miR-306-3p	miR-994-3p, 5p
miR-1007-3p	miR-317-3p	miR-4976-5p	miR-13b-3p	miR-318-3p, 5p	miR-995-3p
miR-1011-3p	miR-3-3p	miR-929-5p	miR-275-5p	miR-4917-3p	miR-996-3p, 5p
miR-274-5p	miR-375-3p, 5p	miR-9a-3p, 5p	miR-282-3p	miR-79-3p, 5p	miR-998-3p
***Males only (21)***			miR-2a-1-5p	miR-92a-5p	miR-9c-3p
miR-1003-3p	miR-263b-3p	miR-7-3p	***Males only (45)***		
miR-1010-3p	miR-2a-2-5p	miR-954-3p, 5p	miR-1014-3p	miR-4977-3p	miR-978-3p, 5p
miR-137-3p	miR-2a-3p	miR-969-5p	miR-125-3p	miR-4985-5p	miR-979-3p, 5p
miR-13b-3p	miR-2b-2-5p	miR-988-3p	miR-2498-3p, 5p	miR-8-3p, 5p	miR-980-5p
miR-14-5p	miR-2b-3p	miR-995-3p	miR-2499-3p, 5p	miR-929-5p	miR-982-3p
miR-219-3p	miR-307b-5p	miR-998-3p	miR-274-5p	miR-9369-3p, 5p	miR-985-3p
miR-2535b-3p	miR-4952-3p		miR-303-5p	miR-9370-5p	miR-986-3p
			miR-307b-3p	miR-972-3p	miR-992-3p
			miR-31a-3p	miR-973-3p, 5p	miR-997-3p
			miR-33-3p	miR-974-3p, 5p	miR-iab-4-5p
			miR-375-3p, 5p	miR-975-5p	miR-iab-8-3p
			miR-4966-3p, 5p	miR-976-3p	
			miR-4976-5p	miR-977-3p, 5p

**Table 4:** miRNAs with sex-biased expression in both body-parts (whole fly), or in the head-thorax or abdomen only. Numbers in paratheses are the total miRNAs in each category, inclusive of 3p and 5p strands where both are differentially expressed.

Female biased miRNAs		Male biased miRNAs		
*Whole fly (12)*		*Whole fly (8)*		
miR-286-3p	miR-956-5p	miR-1006-3p	miR-252-5p	miR-2c-3p
miR-2b-2-5p	miR-989-3p	miR-10-5p	miR-263a-5p	miR-993-3p
miR-308-3p, 5p	miR-994-5p	miR-133-3p	miR-263b-3p	
miR-318-3p	miR-9b-3p	***Abdomen only (97*** *)*		
miR-92a-3p	miR-9c-5p	let-7-5p	miR-314-3p	miR-970-3p, 5p
miR-92b-3p		miR-1000-5p	miR-315-5p	miR-972-3p
***Abdomen only (42*** *)*		miR-100-3p	miR-316-5p	miR-973-3p, 5p
bantam-3p	miR-311-3p, 5p	miR-1004-3p	miR-317-3p	miR-974-5p
miR-1003-3p	miR-312-3p, 5p	miR-1013-3p	miR-31a-3p, 5p	miR-975-5p
miR-1010-3p	miR-313-3p, 5p	miR-1015-3p	miR-31b-5p	miR-976-3p
miR-11-5p	miR-318-5p	miR-10-3p	miR-3-3p	miR-977-3p, 5p
miR-13b-2-5p	miR-7-5p	miR-12-3p	miR-34-3p, 5p	miR-978-3p, 5p
miR-13b-3p	miR-79-3p	miR-125-3p, 5p	miR-375-3p, 5p	miR-979-3p, 5p
miR-184-3p	miR-92a-5p	miR-12-5p	miR-4919-3p	miR-980-5p
miR-2489-3p	miR-9372-5p	miR-1-3p	miR-4966-3p, 5p	miR-981-3p
miR-275-3p	miR-988-3p	miR-2498-3p, 5p	miR-4976-5p	miR-982-3p, 5p
miR-279-3p	miR-989-5p	miR-2499-3p	miR-6-3p	miR-983-3p, 5p
miR-282-3p, 5p	miR-994-3p	miR-263a-3p	miR-8-3p, 5p	miR-984-3p, 5p
miR-284-3p, 5p	miR-995-3p	miR-274-5p	miR-87-3p	miR-985-3p
miR-2a-1-5p	miR-996-3p, 5p	miR-276b-3p	miR-929-5p	miR-986-3p
miR-2a-3p	miR-998-3p, 5p	miR-277-3p, 5p	miR-9369-3p, 5p	miR-987-5p
miR-2b-3p	miR-999-3p	miR-278-3p	miR-9370-5p	miR-991-3p
miR-306-3p, 5p	miR-9b-5p	miR-281-1-5p	miR-959-3p, 5p	miR-992-3p
miR-310-3p	miR-9c-3p	miR-281-2-5p	miR-960-3p, 5p	miR-997-5p
*Head-thorax only (12)*	miR-281-3p	miR-961-3p, 5p	miR-9a-3p, 5p	
miR-281-1-5p	miR-375-3p	miR-303-5p	miR-962-3p, 5p	miR-iab-4-3p
miR-283-5p	miR-8-3p	miR-304-3p, 5p	miR-963-5p	miR-iab-8-5p
miR-314-3p, 5p	miR-956-3p	miR-307a-5p	miR-964-3p, 5p	
miR-316-5p	miR-958-3p, 5p	miR-307b-3p	miR-969-3p	
miR-33-5p	miR-980-3p	***Head-thorax only (9)***		
		miR-1017-3p	miR-210-5p	miR-990-5p
		miR-124-3p, 5p	miR-932-3p	miR-998-3p
		miR-190-3p	miR-957-3p	

### Mating-Responsive miRNAs

Significantly (padj <0.05) differentially expressed miRNAs in response to mating were found in the Ab tissue of both males and females (Table 1, Supplementary Table S8a–d). In the female Ab, three miRNAs were significantly upregulated in response to mating (miR-14-3p, miR-997-5p, and miR-184-5p), and one miRNA was downregulated (miR-286-3p). In the male Ab, both strands of miR-927 were significantly downregulated in response to mating. No miRNAs were significantly DE in the HT of either sex.

### mRNA-miRNA interactions

A number of mating-responsive mRNA targets of the differentially expressed miRNAs were identified, including genes which were dysregulated in the opposite direction of the targeting miRNA (*i.e*. an upregulated target of a downregulated miRNA, or a downregulated target of an upregulated miRNA) (Supplementary Table S9). Of the upregulated miRNAs, only miR-184-5p in the female abdomen was linked to any significantly downregulated targets, with one gene identified. For downregulated miRNAs, miR-286-3p of the female abdomen contains four significantly upregulated targets. For the male abdomen, there were 50 and 114 significantly upregulated targets for the downregulated miRNAs miR-927-3p and miR-927-5p, respectively. Six of these significantly upregulated genes are predicted to be targeted by both miR-927-3p and miR-927-5p, namely *Rpl37a*, *CG5707*, *Slh*, *twi*, *Su(w[a])* and *Nop60B*. Network visualisations of miR-927-3p and miR-927-5p interactions with all putative, mating-responsive mRNA targets (regardless of direction of differential expression) revealed the extensive targeting of both upregulated and downregulated mRNAs, including an additional 13 downregulated mRNAs targeted by both strands of miR-927 (Supplementary Fig. S3,a,b). To test whether the predicted targets of miR-927 were functionally linked, we conducted a GO enrichment analysis on the upregulated predicted targets of each strand of miR-927. However, the analysis did not yield any enriched terms with an FDR q-value > 0.05.

When comparing predicted target and non-target transcripts of each differentially expressed miRNA for each comparison (e.g. mated versus virgin female Ab), the Kolmogorov-Smirnov analyses did not show differences between the fold change distributions of predicted targets and non-targets (Supplementary Fig. S6). Similarly we found no evidence for the overrepresentation of miR-184-5p, miR-286-3p or miR-927 targets amongst the corresponding set of mating-responsive mRNAs, when compared to all mRNAs (Fisher’s Exact Test).

## Discussion

We tested two major predictions (i) that there are significant changes to the expression of coding and regulatory non-coding genes following mating in both sexes, and (ii) that the mode and nature of PMR gene expression profiles of each sex are markedly different. The results supported both predictions and revealed significant insights into sex-specific functional variation in post-mating responses. Our data showed strong signatures of mating-responsive gene expression profiles that were unique, and spatially distinct, in each sex. In females, differential expression was generally of larger magnitude than in males. Gene expression in the female head-thorax was radically altered by mating, with 2040 genes showing differential expression of substantial magnitude, while 125 genes responded to mating in female abdomens. In contrast, in males there were no mating-responsive expression changes in the head-thorax at all under the same significance criteria, whereas male abdomens showed differential expression in 2068 genes.

The large number of DE genes in the female head-thorax and male abdomen is consistent with known PMR activity and phenotypes in those different body-parts. For example, the receipt of the ‘sex peptide’ seminal fluid protein from males during mating causes neurological changes in the female brain that affect feeding behaviour, sleep patterns, sexual receptivity and aggression levels^14–16^. Hence even if the primary site of Sfp receipt is within the female reproductive tract in the abdomen, Sfps can cause many changes in other parts of the body by binding to receptors located in the nervous system including the brain^62^. Our knowledge of PMR phenotypes in males is limited, but biological processes known to be affected by mating are located within the abdomen, namely the replenishment of ejaculate components and morphological changes to the ejaculatory duct^31,33^. The low numbers of DE genes seen in the female abdomen and male head-thorax suggests there is lower activity of biological processes in those body-parts following mating, or a low requirement for active *de novo* gene transcription. However, it is also possible that gene expression changes in different tissues and cell types within the major body parts tested are occurring, but counteracting one another. The gene expression patterns we describe are supported by specific validated genes reported from other studies. Previous transcriptomic studies on females varied considerably in the numbers of DE genes detected in response to mating, from just 38^43^ to over 2000^44^ in whole females assayed a few hours after mating. One study^49^ also found a ten-fold difference in the numbers of mating-responsive genes in the spermatheca compared to the seminal receptacle, indicating that tissues with related functions can also show distinctive responses.

We also observed sex-specific functional enrichment amongst mating-responsive genes. For example, in male abdomens, the predominant response was an upregulation in genes associated with protein folding, localization and processing through the Golgi apparatus and endoplasmic reticulum. Genes encoding signal recognition particles (SRP), SRP receptors, translocation channel proteins and p24 family proteins^63^ were upregulated, as well as genes encoding coatomer-proteins that form COPI and COPII vesicles^64,65^. This sex-specific response implies an increase in the production of secreted or transmembrane proteins, and is consistent with male replenishment of Sfps that become depleted following mating^31^. Most of the secretory pathway transcripts were upregulated less than two-fold in mated versus virgin males, which suggests a complex coordinated and on-demand regulation machinery for the production of Sfp proteins. In female abdomens, the transcription of genes encoding immune effectors was elevated following mating, consistent with previous observations^43,46,47,50^. The significance of this robust response is not yet clear but may stem from either the transfer of pathogens through mating, damage to the female genital tract, or induction by ejaculate proteins^20,66^.

As well as sex-specific responses, a core set of shared genes were differentially expressed in both male and female abdomens. Among these was an over-representation of ribosomal protein (RP) genes and genes involved in the electron transport chain. This implies that both sexes have an increased requirement for translation and energy generation following mating. In males, increased translation in the abdomen is consistent with the replenishment of Sfps. Indeed, a previous study reported a burst of ribosome synthesis in the accessory glands (the main site of Sfp production) of males between 30 minutes and 6 hours following mating^67^. Increased translation in mated female abdomens may be required for egg activation and the progression of vitellogenic oocytes, requiring the translation of maternal mRNAs and enhanced yolk protein synthesis, respectively^68,69^.

Interestingly, some mating-responsive processes that were upregulated in the abdomen of both sexes were downregulated in the female head-thorax. Indeed, downregulated genes constituted the majority of DE genes in the female head-thorax, in direct contrast to all other comparisons, in which DE was generally due to the upregulation of genes in response to mating. Reduced expression of RP genes and genes associated with energy generation in the head-thorax, and elevation of those same genes in the abdomen, is suggestive of a mating-induced ‘switch’ in tissue-specific resource allocation. This could reflect a compensatory mechanism to counterbalance the increased demand for energy and translation in the abdomen, an idea that would be interesting to test.

It can be somewhat difficult to directly compare transcriptomic studies across different laboratories, given the variance in experimental design, diet, fly strain, tissue, transcriptomic methods and analysis. Nevertheless, there are some interesting contrasts to explore with existing studies of the transcriptomic responses of female *D. melanogaster* to mating^42–49,51^. To minimise confounding variation, we compared our data with studies that had used similar time points and tissues and in general, there was good overlap. Specific genes were robustly differentially expressed in response to mating across multiple studies. These included *fit*, *CG31324* and *Send2* which were upregulated in response to mating in this and in five other studies. *Send2* encodes the serine protease spermathecal endopeptidase 2 which, along with *Send1*, is exclusively expressed in secretory cells of the female spermatheca^70^. Although the exact function of Send2 itself is unknown, products of the spermathecal secretory cells are required for the recruitment of sperm for storage, and sustained egg laying^70^. The gene product of *CG31324*, which was also consistently upregulated in the mated female abdomen, is currently unknown. The product of *fit* is associated with feeding behaviour. In females, it is downregulated in starvation conditions^71^ and acts as a negative feedback regulator to suppress the intake of protein-rich food^72^. Mated females have an increased appetite^14^, and a preference for protein-rich food when compared to virgins^73^. Therefore the upregulation of *fit* following mating may be triggered by the protein-component of an increased food intake.

Our data revealed that post-mating changes in both sexes have the potential to be regulated by small RNA molecules, as has been reported previously for females^46,57^. The pluripotentiality of miRNA targeting allows multiple genes to be regulated simultaneously under the influence of miRNA ‘hubs’^24^. This facilitates the coordinated expression of genes with related functions in response to an appropriate single stimulus, such as mating. At the most stringent significance threshold, we identified four miRNAs that differed in expression between virgin and mated female abdomens, and two in male abdomens. Both the 3p and 5p strands of miR-927 were downregulated in males following mating. In support of a role for this miRNA in regulating reproductive processes, deletion of miR-927 in male *D. melanogaster* is reported to reduce adult fertility^58^. Interestingly, both strands of miR-927 were also among the most significantly downregulated miRNAs in the female abdomen (albeit below the padj threshold of 0.05), suggesting that this miRNA may play a role in the regulation of post-mating responses common to both sexes.

Of the four miRNAs that were significantly differently expressed in mated females, the two with the greatest fold change were miR-184-5p and miR-997-5p. Increased expression of miR-184 is consistent with the essential role of this miRNA in the regulation of oogenesis. Females lacking miR-184 show an age-progressive failure to produce eggs^74^ and their fecundity is unaffected by the presence of sex peptide^57^. Interestingly, overexpression of miR-184 in both sexes causes a severe reduction in lifespan^75^ and this may offer clues to the mechanisms underlying reduced lifespan in mated females. miR-997 was completely absent in virgin females and in female head-thorax tissue, but was detectable in female abdomens following mating. Notably, in males, miR-997 expression was also restricted to the abdomen, but is stably expressed regardless of mating status. One possibility is that miR-997 is expressed solely by males and transferred to females during mating. Extracellular miRNAs can be transported stably within microvesicles, which are released into the ejaculate by secondary cells of the male accessory gland^76,77^. Once in the female, miRNAs contained within the microvesicles have the potential to target female mRNA molecules, and thus alter female post-mating responses^76^.

The miRNA-mRNA interaction analysis identified a number of genes that were differentially expressed in the opposite direction to significantly upregulated or downregulated miRNAs, indicating a potential response of the coding transcriptome to miRNA differential expression after mating. However, global differences in expression between all predicted targets and non-targets of differentially expressed miRNAs were not observed. A potential explanation is that miRNA differential expression influences physiological change by mediating the repression of a restricted set of predicted targets. The signal for this type of repression would be obscured in a global analysis of all predicted targets and nontargets. The global correlation analysis of mating-responsive miRNAs and mRNAs also revealed that a number of differentially expressed mRNAs in the male abdomen had the potential to be targeted by miR-927-3p or 5p strands. Of the mRNA targets that were expressed in the opposite direction to miR-927, at least 38 are described as having a role in developmental processes, although there was no overall significant enrichment of functional terms, suggesting that putative targets of miR-927 have diverse functions. Interestingly, a number of DE mRNAs were predicted to be targets of both strands of miR-927, opening up the possibility that the two mature miRNAs are acting cooperatively to mediate repression.

## Conclusion

Our results provide the first direct comparison of the transcriptomic responses of male and female *Drosophila* to mating, and the first comparison of mating-responsive miRNAs in both sexes in any species. Our data reveal that there were marked sex- and body part-specific responses to mating, in profiles of mRNAs and miRNAs in *D. melanogaster*. However, some transcriptional responses were also shared by males and females. There were also sex-specific differences in the magnitude of gene expression changes, with females generally showing a greater magnitude of DE. In addition, while many of the same genes were differentially expressed between body-parts and sexes, the direction of DE of these genes was sex- or tissue-specific. Taken together, our results show that while both males and females invest in enhanced protein and energy production in the abdomen, males have a much broader response than females, and additionally invest in the production of secretory protein pathway components. In contrast, in the head-thorax, females showed the greatest transcriptional response through the downregulation of both small- and macro-molecule metabolism and energy production, while the transcriptional profile of males remained largely unchanged. Our results reveal the extent of quantitative and qualitative variation in sex-specific responses to mating and highlight novel potential roles for regulatory molecules in shaping the expression of sex differences.

## Materials and Methods

### Sample preparation

Wildtype *D. melanogaster* flies were from a large laboratory population originally collected in the 1970s in Dahomey (Benin). Flies were reared on standard sugar yeast (SY) medium (100 g brewer’s yeast powder, 50 g sugar, 15 g agar, 30 ml Nipagin (10% w/v solution), and 3 ml propionic acid, per litre of medium) in a controlled environment (25 °C, 50% humidity, 12:12 hour light:dark cycle). Larvae were raised at a standard density of 100 per vial (glass, 75 × 25 mm, each containing 7 ml SY medium). Male and female adults were separated within 6 hours of eclosion using ice anaesthesia and stored in single sex vials at a density of 10/vial for 6 days. For the mated treatment, a single male was placed with a female and the time of mating was recorded. Immediately after mating the male was removed to a separate vial to prevent further matings. All mated flies were then flash frozen at 3 hours after start of mating in liquid N_2_. For the virgin treatment, males and females were housed individually in vials for ~3–4 hours before flash freezing. Frozen flies were stored at −80 °C until use. The sample size for each treatment was 50 males and 50 females. The entire experiment was repeated exactly, using fresh egg collections to generate two biological replicates. Therefore, in total 16 samples were generated: 2 sexes × 2 treatments (mated/virgin) × 2 body parts (HT and Ab) × 2 replicates.

### RNA extraction

To prepare tissue for RNA extraction, 50 flies from each sex, treatment and biological replicate were separated into HT and Ab tissues on dry ice, and the body parts were then pooled for RNA extraction (note that both body parts were intact, and thus the Ab contained the germline). Tissues were disrupted by grinding under liquid nitrogen, then total RNA was extracted using the miRvana miRNA isolation kit (Ambion, AM1561), according to the kit protocol. RNA was eluted in RNA storage solution (1 mM sodium citrate, pH 6.4 +⁄− 0.2, Ambion). Samples were DNase treated to remove residual genomic DNA (Ambion Turbo DNA-*free* kit, AM1907). RNA was assessed for quantity and quality using a NanoDrop 8000 spectrophotometer.

### Library construction and sequencing

The 16 samples were sent to the Earlham Institute provider (Norwich Research Park, UK) for mRNA and sRNA library construction, and sequencing. Libraries were constructed using the Illumina TruSeq kit. For the sRNA libraries, a modified ‘blocking oligo’ was also used to preclude adapter ligation to the highly abundant 30 nt 2S rRNA^60^. Non-directional, single end RNA-seq was conducted using the Illumina HiSeq. 2500 platform with 50nt read length.

### Sequencing analysis

Kallisto version 0.46.0^78^ was used to pseudoalign reads to the Berkeley *Drosophila* Genome Project 6 (BDGP6) cDNA sequences downloaded from Ensembl (release 89^79^). A kallisto index was created using the “kallisto index” command (k-mer size 31). Kallisto quant was used to obtain transcript count estimates and parameters were set to include 100 bootstrap samples and to perform sequence bias correction. Transcript to gene mappings were obtained using biomaRt^80^ and transcript counts were aggregated in Sleuth (version 0.28.1)^81^ before calling pairwise differential expression between mated and virgin samples of the same body part and sex. Small RNA reads were converted from FASTQ to FASTA format and then processed to trim sequencing adaptors using a custom Perl script (avalible in the Supplementary Material) recognising the first 8 bases of the adapter sequence (‘TGGAATTC’). Trimmed reads were then aligned to miRBase (v22.0) *D. melanogaster* mature miRNA sequences using PatMaN^82^ (parameters -e 0 -g 0). A custom Perl script (see Supplementary Material) was used to parse the alignment files and generate an aligned read count table across all samples. DESeq2 (version 1.14.1)^59^ was used for normalisation of counts between samples and calling differentially expressed miRNAs.

### miRNA target prediction

Prediction of miRNA target sites were conducted using the TargetScan algorithm (version 4.1 – ‘TargetScanS’)^83^, which predicted targets on the basis of complementarity of the 3′ untranslated region (3′UTR) to the mature miRNA seed sequence^83,84^. In order to run TargetScan, using custom shell scripts, mature miRNA sequences were downloaded from miRBase, filtered for *D. melanogaster* miRNAs, and processed to produce a tab-delimited three column file, with columns sequentially referring to miRNA name, miRNA seed sequence (*i.e*. nucleotides 2–8 of the miRNA from the 5′ end), and NCBI taxonomic ID (*i.e*. ‘7227’ for *D. melanogaster*). The R (v3.5.1)^85^ biomaRt package (v2.38.0)^80,86^ was used for the download of *D. melanogaster* 3′UTR sequences necessary for the running of TargetScan, along with transcript-gene mappings: The *useMart* function was used to select the Ensembl mart. For the selected mart, the *useDataset* function was used to select *D. melanogaster* ensembl gene models for release 89 of Ensembl. Afterwards the *getBM* function was used to extract stable gene and transcript ids, external gene names and 3′UTR sequences for all *D. melanogaster* transcript models. Otherwise default parameter values were used when calling biomaRt functions. For each gene model, the transcript splice-isoform (denoted by the ensembl transcript ID) with the longest annotated 3′UTR was designated as being representative for that gene. In cases where a gene model possessed multiple transcript isoforms corresponding to the maximum 3′UTR length for that gene, one transcript isoform was selected at random. Gene models in which none of the corresponding transcript models possessed an annotated 3′UTR sequence was not used for miRNA target prediction with TargetScan. For use with TargetScan, 3′UTR data were deposited in a three-column tab-delimited text file sequentially containing an identifier column containing the ensembl gene ID, ensembl transcript ID, and the external gene name; a column containing the *D. melanogaster* NCBI taxonomic ID (*i.e*. ‘7227’), and a final column containing the 3′UTR sequence. TargetScan was then subsequently executed with the 3′UTR data file and the previously described miRNA data file.

### Comparison of miRNA predicted targets and nontargets

Transcript expression data was pre-processed before statistical testing: The mean average relative abundance for each mRNA, in units of normalised transcripts per million (TPM)^87^, was computed from both replicates for each sample type (*e.g*. female abdomen). mRNA with average normalised TPM values equal to zero were discarded for each sample type and not used for further analysis. A pseudocount (AKA offset) of 1 was added to all remaining average normalised TPM values. Log_2_ fold change values were computed from offset average normalised TPM values for each individual differential expression analysis (e.g. virgin male abdomen *vs*. mated male abdomen). The log_2_ fold change was subsequently used as a metric of the magnitude of mRNA differential expression between conditions. Exploratory Data Analysis, in which cumulative plots of upregulated, downregulated and not differentially expressed genes were constructed with respect to 3′UTR length and 3′UTR predicted target site frequency, indicated that 3′UTR length was a potential confounding variable when examining mRNA dysregulation between the virgin and mated conditions (Supplementary Figs S2ab and S4ab). A simulation, in which TargetScan was ran as described previously, but using 401 randomly generated miRNA seed sequences instead of 401 *D. melanogaster* miRNA seed sequences, and with subsequent cumulative plot construction with respect to 3′UTR target site frequency, provided further evidence that 3′UTR length was a confounding variable (Supplementary Fig. S5a,b). In subsequent analyses, a sampling method was used to normalise 3′UTR length for mRNA expression data when comparing predicted target 3′UTRs to predicted nontarget 3′UTRs for any given comparison: A histogram of 3′UTR sequence lengths was constructed separately for predicted target and nontarget datasets, starting from 0, in increments of 200nt, and to a maximum representing the maximum sequence length from both target and non-target datasets. Each break of the two histograms are iterated through, and for each iteration, log fold change values for predicted target and nontarget datasets are subsetted to fall within the 3′UTR sequence length range given by the individual histogram breaks. Within this range, of the target and nontarget log fold change vectors, if vector sizes are unequal, the vector with the largest number of records is sampled to match the number of observations contained within the smaller vector. Log fold change values for both predicted target and nontarget datasets are concatenated for each iteration, in order to create log fold change distributions which are normalised for 3′UTR length. In the case of the Fisher Exact test, an identical sampling procedure is implemented with the exception that transcript identifiers are sampled instead of log fold change values. We investigated whether changes in mRNA expression could be influenced by the changes in expression of differentially expressed miRNAs, by a process of miRNA targeting. Two-sample, one-sided Kolmogorov-Smirnov (KS) tests (using the *ks.test* function of the R *stats* package) were implemented to test for the inequality between miRNA target and miRNA nontarget fold change distributions for a given miRNA, and the one-sided Fisher Exact Test (using the *fisher.test* function of the R *stats* package) to test for an enrichment of the target sites of differentially expressed miRNAs in mRNAs differentially expressed in the opposite direction. For the KS test, the value of the ‘alternative’ parameters was set to ‘greater’ when testing the effect of upregulated miRNAs, and set to value of ‘less’ when testing the effect of downregulated miRNAs. For the Fisher test, the value of the ‘alternative’ parameter was always set to a value of ‘greater’. Otherwise, default parameters were used for both statistical test functions. Fisher Exact and KS tests were also similarly conducted to test for potential combinatorial effects of different pairwise combinations of miRNAs which were differentially expressed in the same direction with predicted target sets designated as those mRNAs with predicted targets for both differentially expressed miRNAs. The false discovery rate (FDR) was set at 0.05, with the Benjamini-Hochberg method used to correct for multiple comparisons^88^. To counteract the stochasticity introduced by the sampling described previously, for each test, p-values were calculated 100 times, and the mean average p-value was taken as being representative.

### Network visualisations

Network visualisations of predicted interactions between differentially expressed coding genes and differentially expressed miRNA for the mated male abdomen were completed using Cytoscape (v3.4.0)^89^. Network visualisations were not completed for other comparisons, which either did not possess any differentially expressed miRNAs, or the number of differentially expressed coding genes predicted to be targeted by differentially expressed miRNAs was judged to be too low for network visualisations to be informative.

### Gene ontology enrichment analysis

GO analyses were conducted using the GOrilla enrichment analysis and visualisation tool using the default parameter settings^90,91^. Unranked target lists of genes were compared to a background of genes for which reads were obtained in our sequencing analysis. Background reference lists were tailored to each sex and body part, to minimise sampling bias^92^. The cut-off for statistical significance was an FDR q-value < 0.05.

### Data archiving

Raw sequencing data for this study is stored at the Sequence Read Archive (SRA) using the BioProject accession: PRJNA521155.

## Supplementary information

Supplementary tables

Supplementary figures and PERL scripts
