# Supplementary material for: Divergence in Transcriptional and Regulatory Responses to Mating in Male and Female Fruitflies
Source: Sci Rep. 2019 Nov 6;9:16100. doi: 10.1038/s41598-019-51141-9 (PMC6834580; doi:10.1038/s41598-019-51141-9)
Supplement: Supplementary file 2 — Supplementary figures and PERL scripts [file 41598_2019_51141_MOESM2_ESM.pdf]

Divergence in Transcriptional and Regulatory Responses to Mating in Male and Female Fruitflies.

Emily K. Fowler, Thomas Bradley, Simon Moxon & Tracey Chapman

Supplementary Figures

Figure S1: sample heat plot showing rep-rep variation.

Figure S2a-b: Cumulative plot with target site frequency on the x-axis. (a) mated male abdomen vs virgin female abdomen (B) mated female head-thorax vs virgin female head-thorax.

Figure S3a-b: Network visualisations of DE miRNAs and predicted interactions for (a) male abdomen global interaction network (b) male abdomen shared target of DE miRNAs.

Figure S4a-b: Cumulative plot with 3'UTR length on the x-axis. (a) mated male abdomen vs virgin female abdomen (b) mated female head-thorax vs virgin female head-thorax.

Figure S5a-b: Cumulative plot with target site frequency on the x-axis. Simulated seed sequences (a) mated male abdomen vs virgin female abdomen (b) mated female head-thorax vs virgin female head-thorax.

Figure S6: Cumulative plot of targets and nontargets of dme-miR-14-3p for male head-thorax. Log2 fold change on the x-axis.

Perl Scripts

Script 1: Adaptor trimming

Script 2: miRNA counts from PatMan

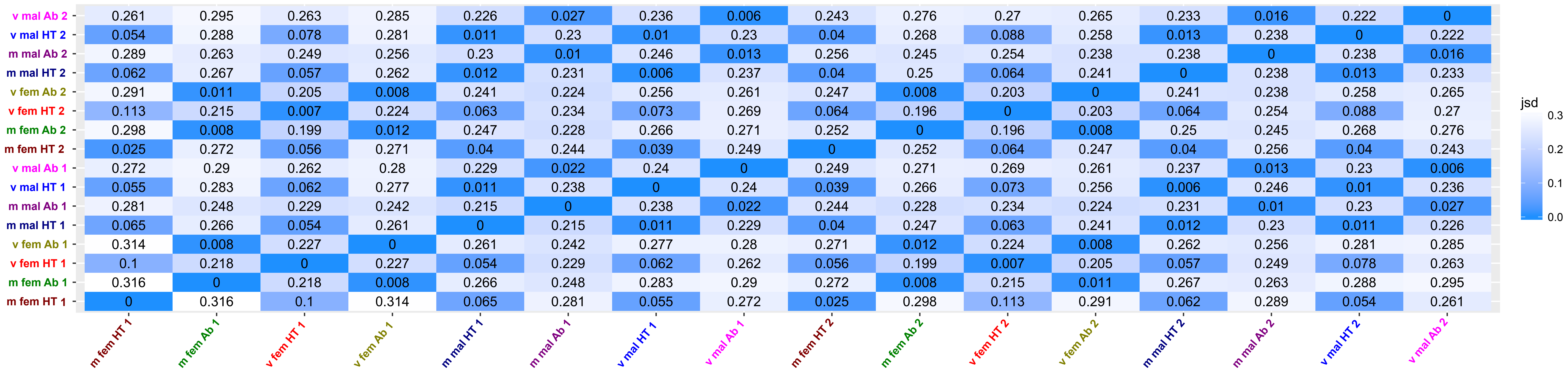
v mal Ab 2 0.261 0.295 0.263 0.285 0.226 0.027 0.236 0.006 0.243 0.276 0.27 0.265 0.233 0.016 0.222 0
v mal HT 2 0.054 0.288 0.078 0.281 0.011 0.23 0.01 0.23 0.04 0.268 0.088 0.258 0.013 0.238 0 0.222
m mal Ab 2 0.289 0.263 0.249 0.256 0.23 0.01 0.246 0.013 0.256 0.245 0.254 0.238 0.238 0 0.238 0.016
m mal HT 2 0.062 0.267 0.057 0.262 0.012 0.231 0.006 0.237 0.04 0.25 0.064 0.241 0 0.238 0.013 0.233
v fem Ab 2 0.291 0.011 0.205 0.008 0.241 0.224 0.256 0.261 0.247 0.008 0.203 0 0.241 0.238 0.258 0.265
v fem HT 2 0.113 0.215 0.007 0.224 0.063 0.234 0.073 0.269 0.064 0.196 0 0.203 0.064 0.254 0.088 0.27
m fem Ab 2 0.298 0.008 0.199 0.012 0.247 0.228 0.266 0.271 0.252 0 0.196 0.008 0.25 0.245 0.268 0.276
m fem HT 2 0.025 0.272 0.056 0.271 0.04 0.244 0.039 0.249 0 0.252 0.064 0.247 0.04 0.256 0.04 0.243
v mal Ab 1 0.272 0.29 0.262 0.28 0.229 0.022 0.24 0 0.249 0.271 0.269 0.261 0.237 0.013 0.23 0.006
v mal HT 1 0.055 0.283 0.062 0.277 0.011 0.238 0 0.24 0.039 0.266 0.073 0.256 0.006 0.246 0.01 0.236
m mal Ab 1 0.281 0.248 0.229 0.242 0.215 0 0.238 0.022 0.244 0.228 0.234 0.224 0.231 0.01 0.23 0.027
m mal HT 1 0.065 0.266 0.054 0.261 0 0.215 0.011 0.229 0.04 0.247 0.063 0.241 0.012 0.23 0.011 0.226
v fem Ab 1 0.314 0.008 0.227 0 0.261 0.242 0.277 0.28 0.271 0.012 0.224 0.008 0.262 0.256 0.281 0.285
v fem HT 1 0.1 0.218 0 0.227 0.054 0.229 0.062 0.262 0.056 0.199 0.007 0.205 0.057 0.249 0.078 0.263
m fem Ab 1 0.316 0 0.218 0.008 0.266 0.248 0.283 0.29 0.272 0.008 0.215 0.011 0.267 0.263 0.288 0.295
m fem HT 1 0 0.316 0.1 0.314 0.065 0.281 0.055 0.272 0.025 0.298 0.113 0.291 0.062 0.289 0.054 0.261
m fem HT 1
m fem Ab 1
v fem HT 1
v fem Ab 1
m mal HT 1
m mal Ab 1
v mal HT 1
v mal Ab 1
m fem HT 2
m fem Ab 2
v fem HT 2
v fem Ab 2
m mal HT 2
m mal Ab 2
v mal HT 2
v mal Ab 2
jsd
0.3
0.2
0.1
0.0

Figure S1 Heat map of sample-to-sample variation for mated (m) or virgin (v), male (mal) and female (fem), head-thorax (HT) or abdomen (Ab). Corresponding biological replicates are in the same colour text.

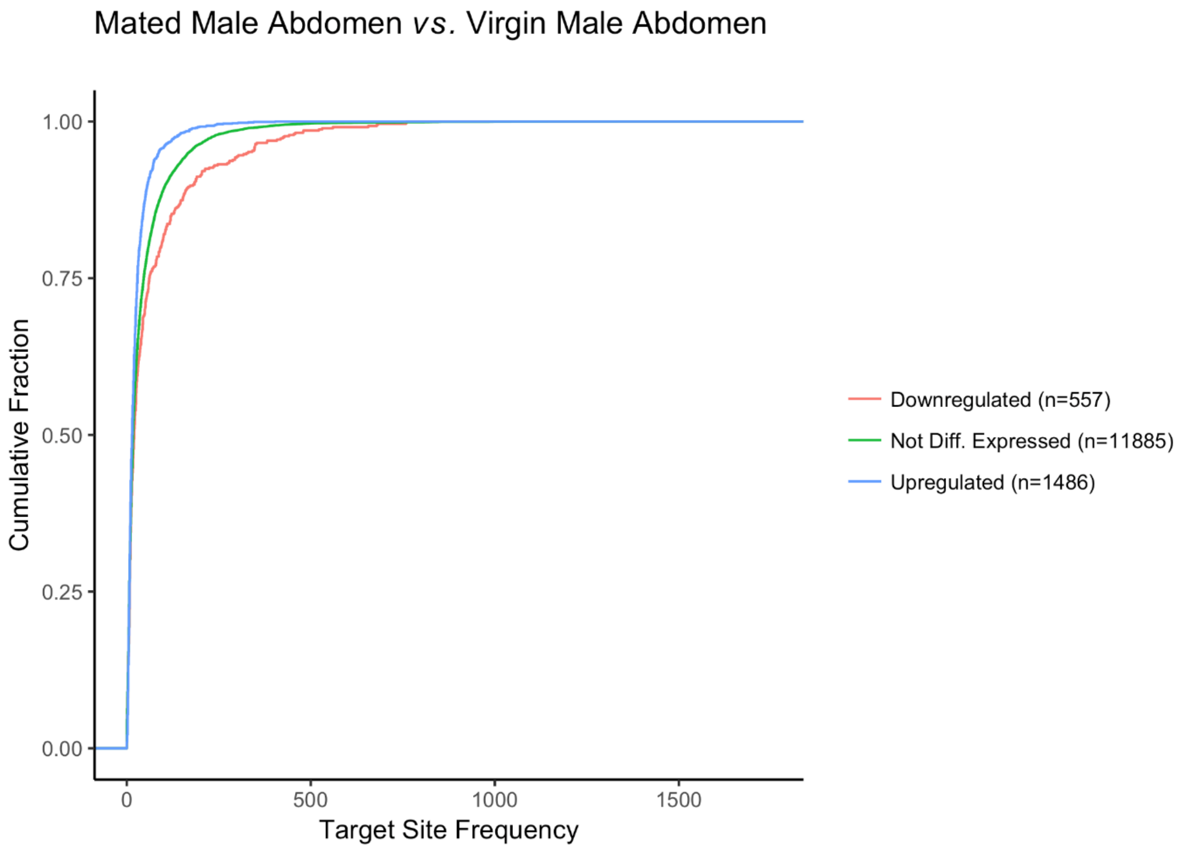

Figure S2a: Empirical cumulative distributions of coding genes which are either upregulated, downregulated or not differentially expressed, with respect to predicted miRNA target site frequency. Comparison: Mated male abdomen vs. virgin male abdomen. This cumulative plot demonstrates that for this comparison, genes downregulated in the mated male abdomen contain a higher proportion of predicted miRNA target sites than genes not differentially expressed in this comparison, which themselves contain a higher proportion of predicted miRNA target sites than coding genes upregulated in the male abdomen.

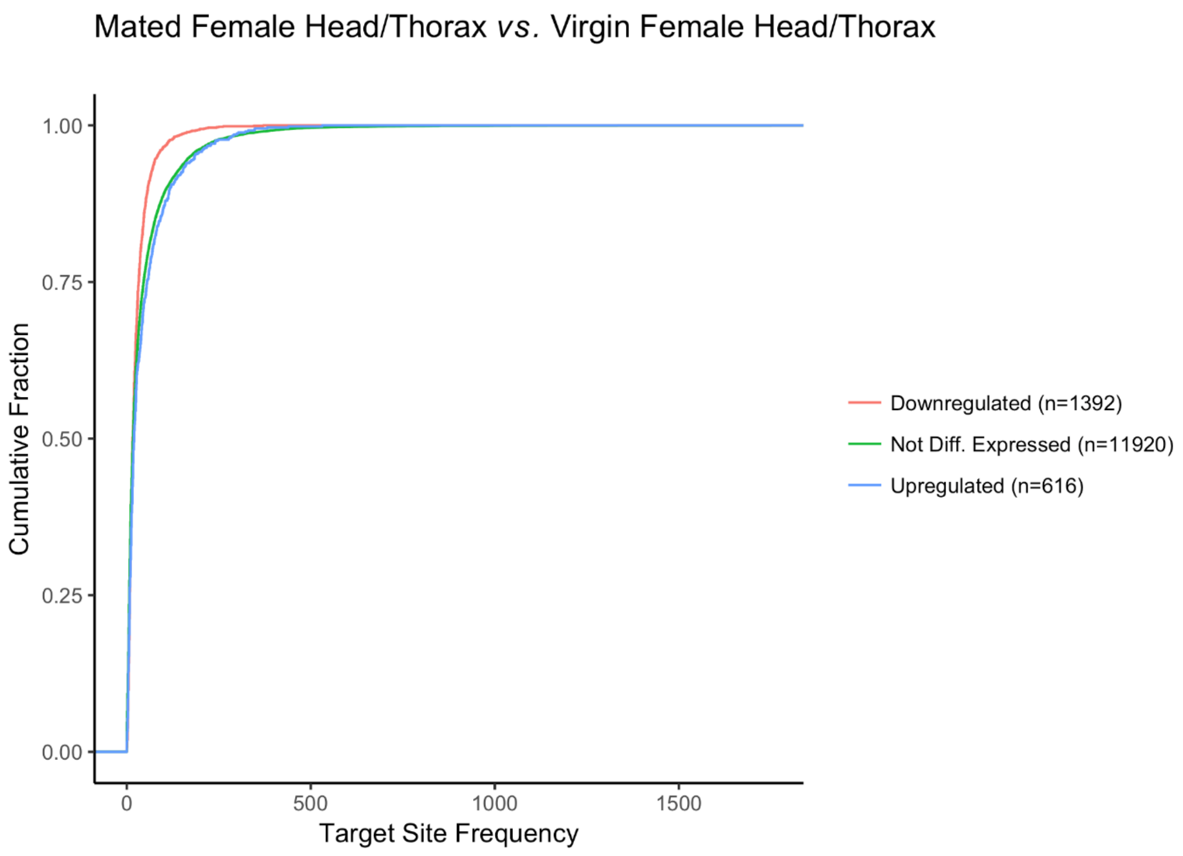

Figure S2b: Comparison: Mated female head/thorax vs. virgin female head/thorax – otherwise, as in **S1a.** This cumulative plot demonstrates that for this comparison, between the mated female head/thorax and the virgin female head/thorax, coding genes which are upregulated, and coding genes which are not differentially expressed in the mated female head/thorax possess a similar proportion of predicted miRNA target sites, which both possess a higher proportion of predicted miRNA target sites than genes which are downregulated in the mated female head/thorax.

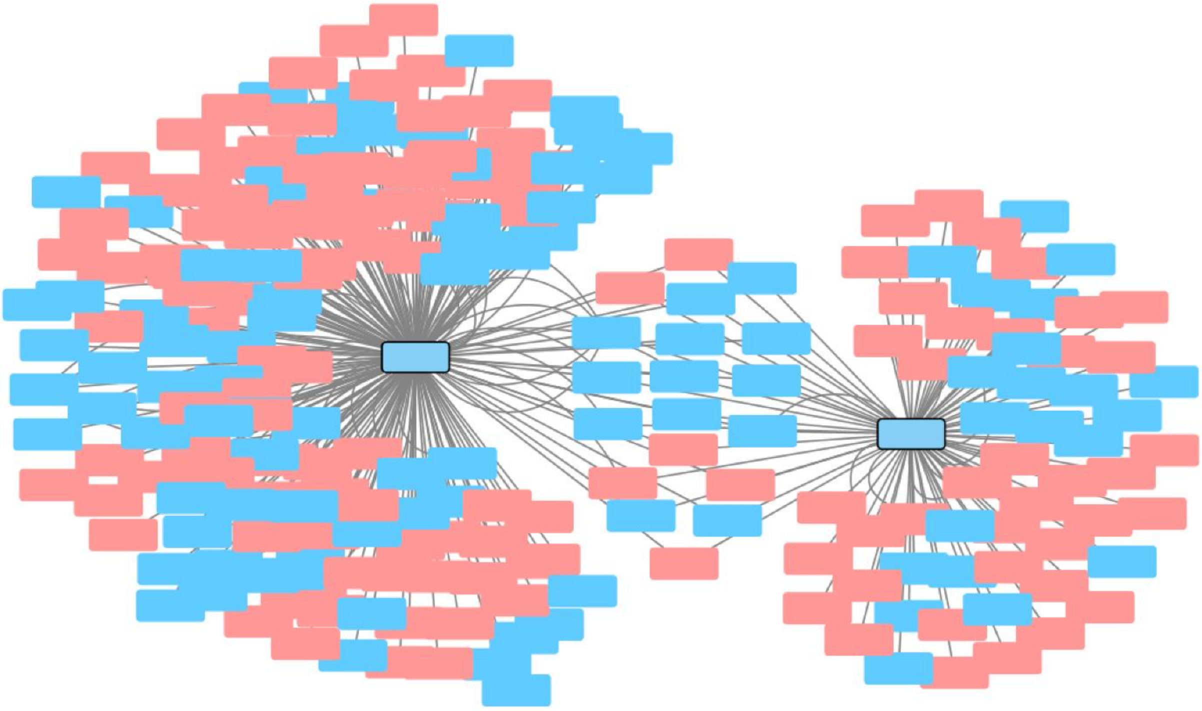

Figure S3a: Network visualisation of predicted miRNA interactions in the male abdomen: Nodes with thick borders denote miRNAs, whilst nodes without thick borders represent coding genes. Nodes coloured pale red denote coding genes upregulated in the mated male abdomen, whilst nodes coloured pale blue denote genes downregulated in the mated male abdomen. Network edges denote a predicted targeting interaction between connected nodes. This visualisation demonstrates the number of shared predicted interactions for differentially expressed miRNAs, the total number of differentially expressed predicted targets of differentially expressed miRNAs, and the proportion of these predicted targets which are either upregulated or downregulated. Network visualisations are not shown for other comparisons which either do not possess any differentially expressed miRNAs, or the number of differentially targets of differentially expressed miRNAs are too low to be informative.

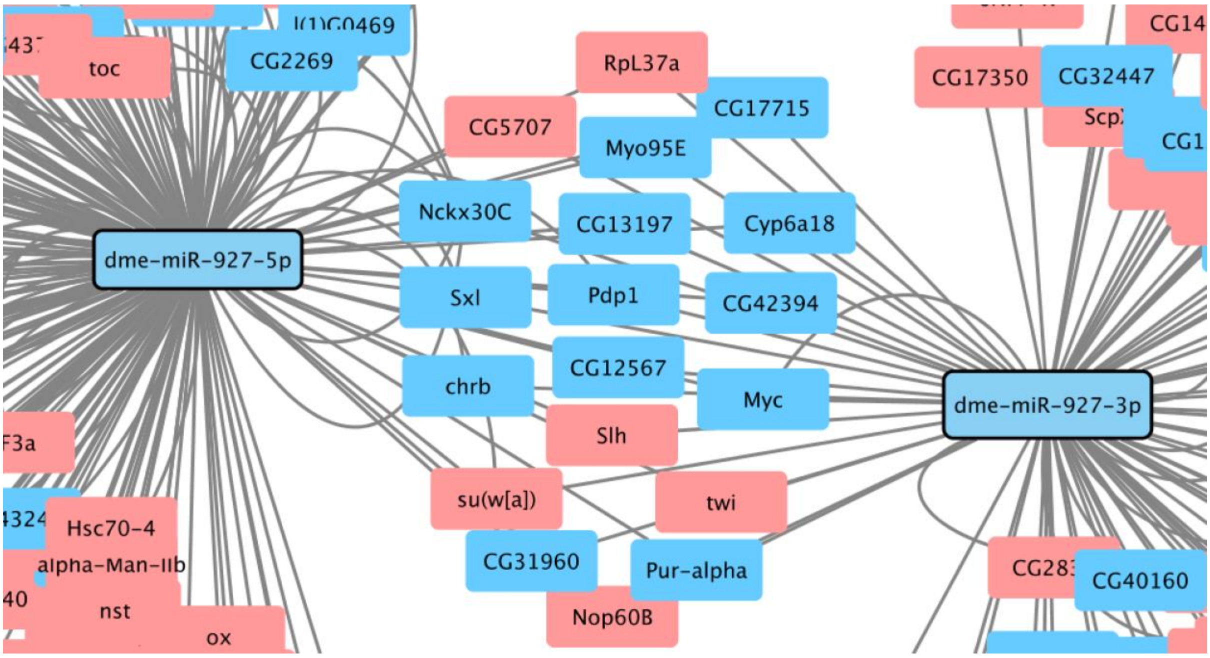

Figure S3b: As in **figure S3a,** though with greater magnification in the central region of that figure, emphasising the shared predicted targets of miRNAs differentially expressed in the mated male abdomen. The shared differentially expressed predicted targets of dme-miR-927-5p and dme-miR-927-3p contain 6 coding genes upregulated, and 13 coding genes downregulated in the mated male abdomen.

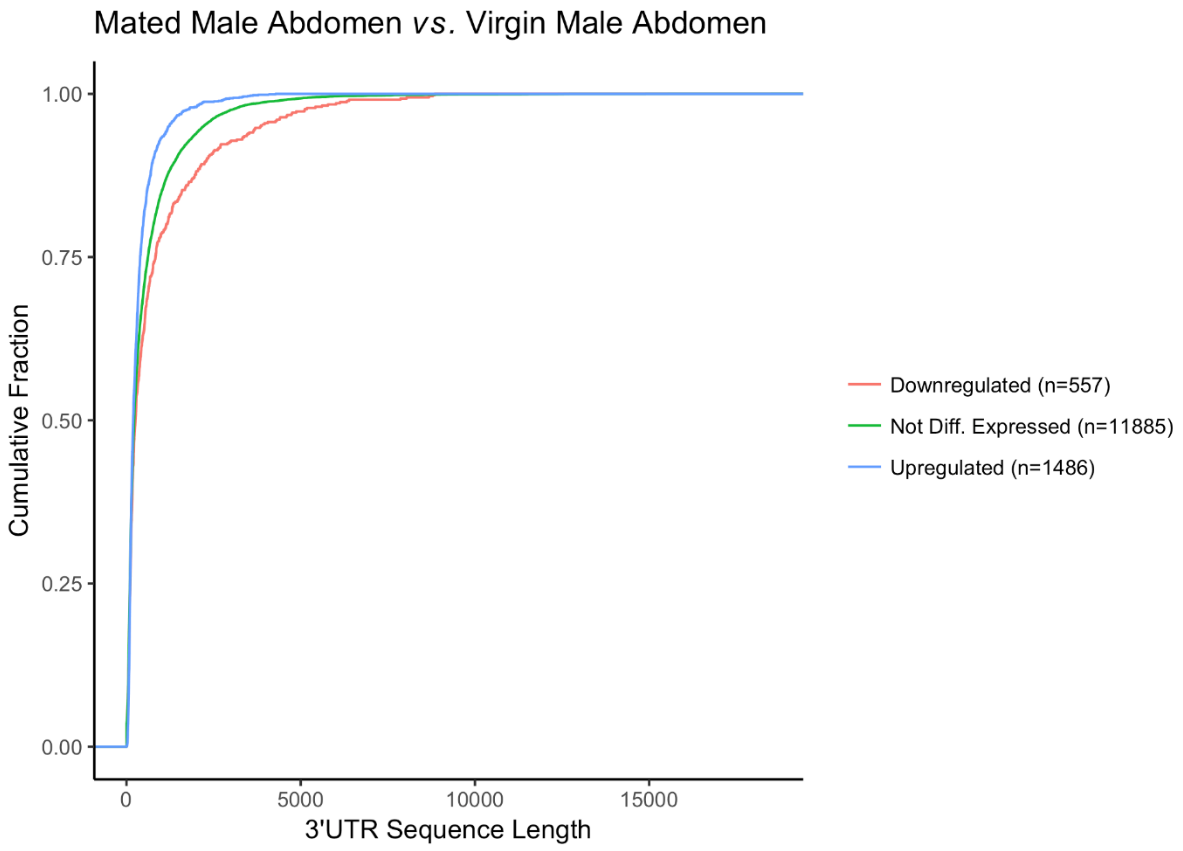

Figure S4a: Empirical cumulative distributions of coding genes which are either upregulated, downregulated or not differentially expressed, with respect to 3'UTR Length. Comparison: Mated male abdomen vs. virgin male abdomen. This cumulative plot demonstrates that coding genes downregulated in the mated male abdomen have proportionately longer 3'UTRs than coding genes which are not differentially expressed in this comparison, which themselves possess proportionately longer 3'UTRs than genes which are upregulated in the mated male abdomen. The pattern of results shown in this cumulative plot is similar to that presented in **figure S2a**, suggesting that 3'UTR length is a potential confounding factor when predicting miRNA targets as part of this analysis.

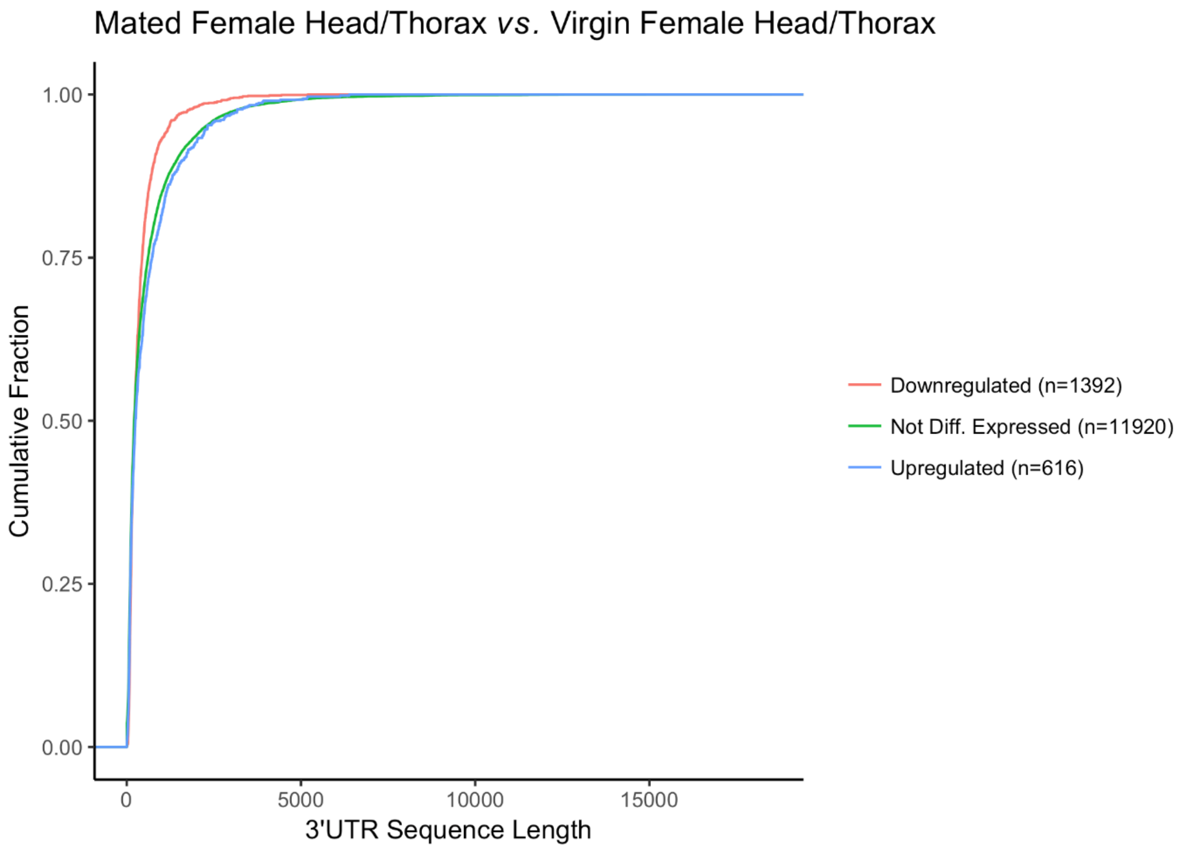

Figure S4b: Comparison: Mated female head/thorax vs. virgin female head/thorax – otherwise, as in **S4a**. This cumulative plot demonstrates that coding genes which are upregulated in the mated female head/thorax possess a similar distribution of 3'UTR lengths to coding genes which are not differentially expressed in the mated female head/thorax, which both possess proportionately longer 3'UTRs than coding genes which are downregulated in the mated female head/thorax. The pattern of results shown in this cumulative plot is similar to that shown in **figure S2b**, suggesting that 3'UTR length is a potential confounding factor when predicting miRNA targets as part of this analysis.

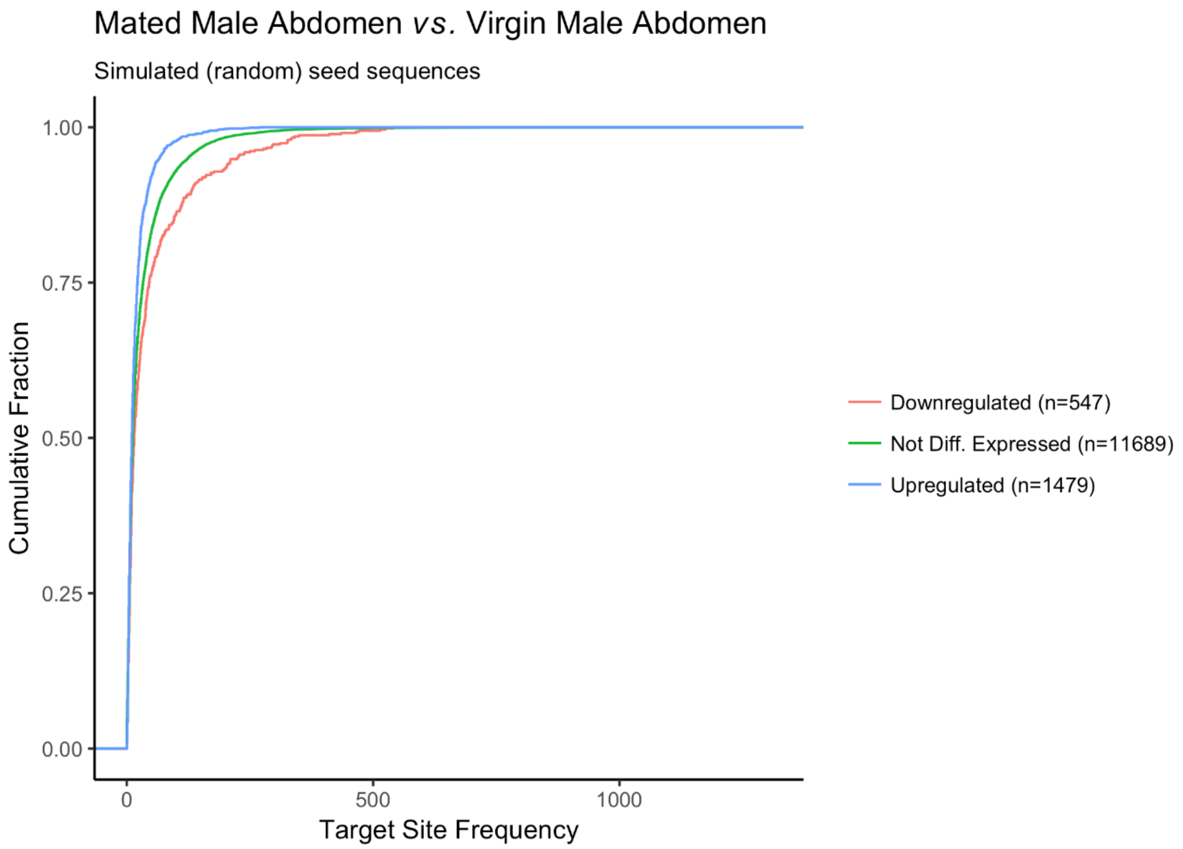

Figure S5a: Empirical cumulative distributions of coding genes which are either upregulated, downregulated or not differentially expressed, with respect to predicted target site frequency of randomly generated miRNA seed sequences. Comparison: Mated male abdomen vs. virgin male abdomen. Predicted target sites were generated by executing the TargetScan algorithm with the *D. melanogaster* 3'UTR set and randomly generated miRNA seed sequences. The cumulative plot demonstrates that genes upregulated in the mated male abdomen possess a proportionately greater number of predicted targets of simulated miRNAs than genes which are not differentially expressed in this comparison, which themselves possess a proportionately greater number of predicted targets of simulated miRNAs than coding genes upregulated in the mated male abdomen. Similar cumulative distribution patterns were observed in **figures S2a** and **S4a**. This analysis demonstrates that the enrichment of predicted miRNA target sites on the 3'UTRs of downregulated coding genes in the mated male abdomen is not likely caused by an evolved adaptation, and the observed pattern of dysregulation of coding genes is unlikely to be caused by the distribution of predicted miRNA target sites on 3'UTRs. This is further evidence that 3'UTR length is a confounding factor when interpreting the results of the differential expression analysis of coding genes as part of this analysis.

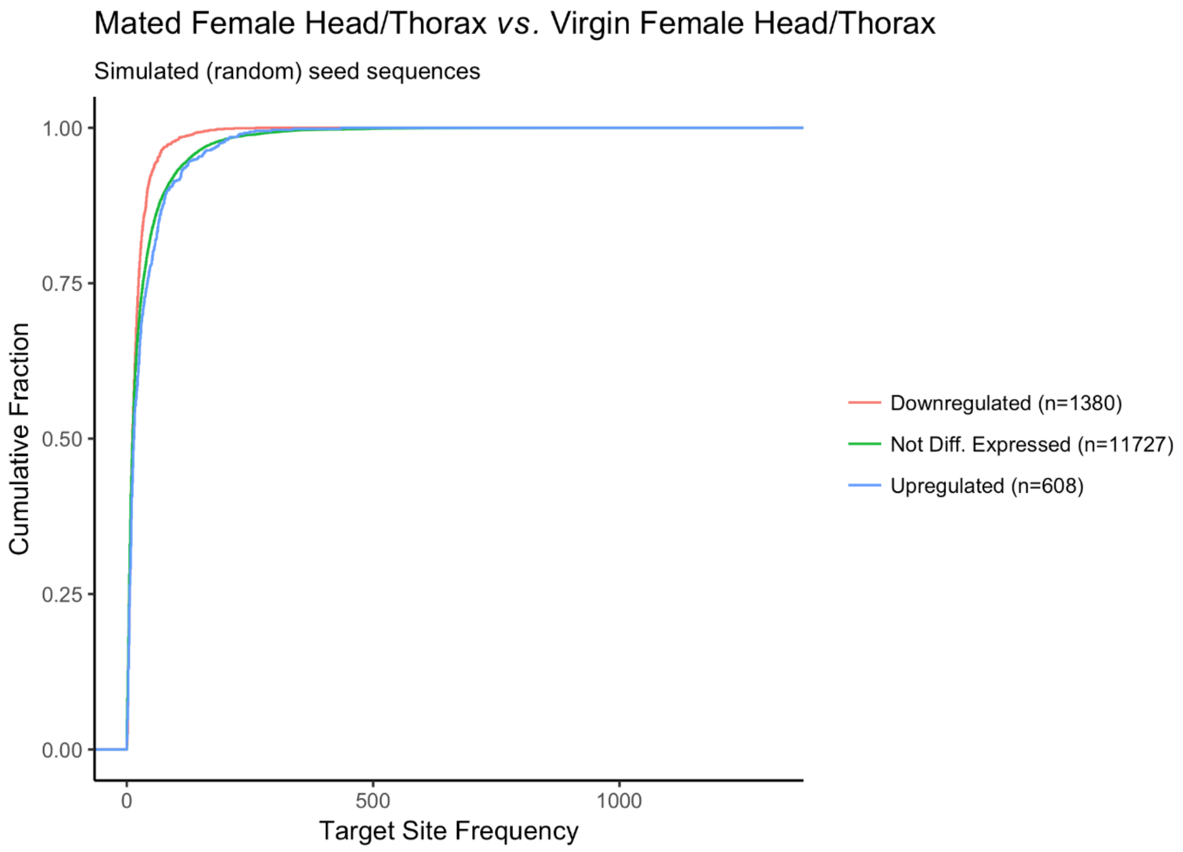

Figure S5b: Comparison: Mated female head/thorax vs. virgin female head/thorax. Otherwise, as in **S5a**. This cumulative plot demonstrates that coding genes upregulated in the mated female head/thorax possess a proportionately similar number of predicted targets of simulated miRNAs as coding genes which are not differentially expressed in this comparison, which both possess a proportionately greater number of predicted targets of simulated miRNAs to coding genes downregulated in the mated female head/thorax. Similar cumulative distribution patterns were observed in **figures S2b** and **S4b**. This analysis demonstrates that the relative depletion of predicted miRNA target sites on the 3'UTRs of downregulated coding genes in the mated male abdomen is not likely caused by an evolved adaptation and the observed pattern of dysregulation of coding genes is unlikely to be caused by the distribution of predicted miRNA target sites on 3'UTRs. This is further evidence that 3'UTR length is a confounding factor when interpreting the results of the differential expression analysis of coding genes as part of this analysis.

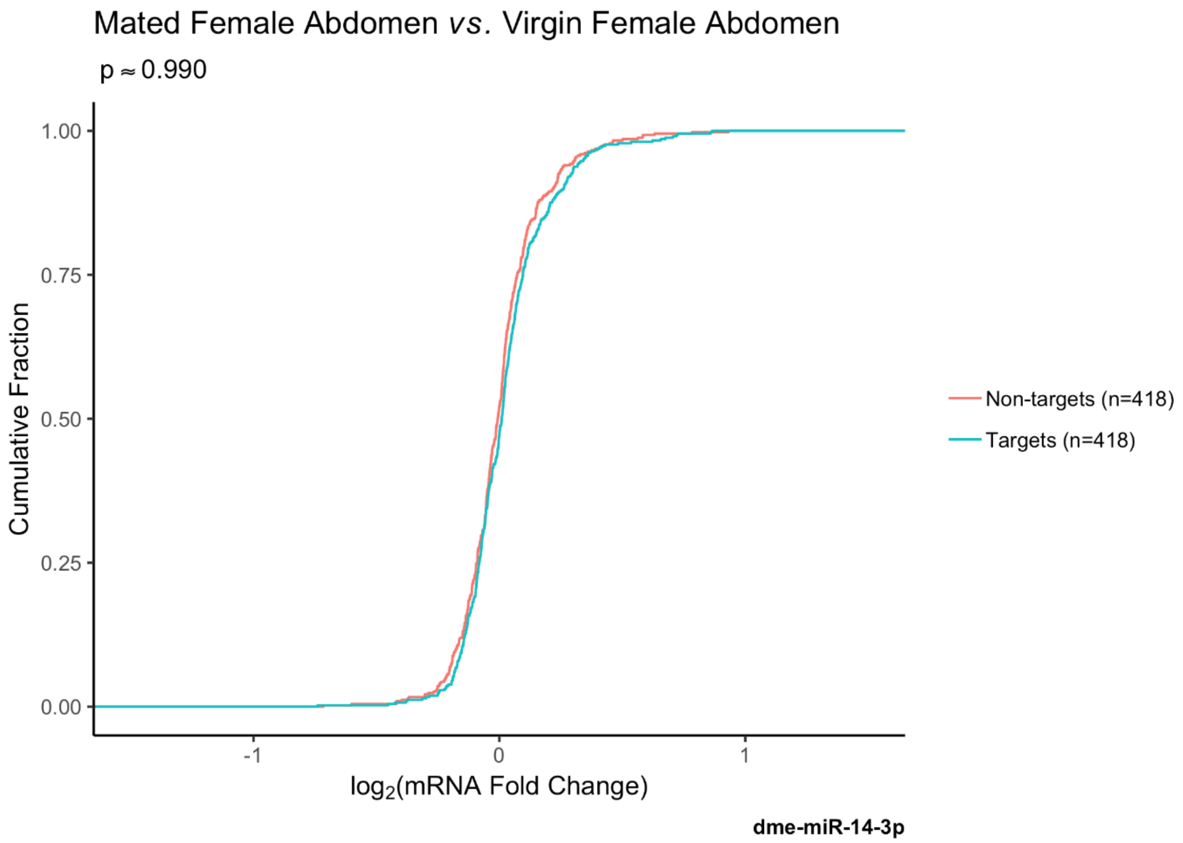

Figure S6: Empirical cumulative distributions of the predicted target and non-targets of dme-miR-14-3p with respect to the $\log_2$ mRNA fold change. dme-miR-14-3p was chosen as a miRNA exhibiting typical behaviour of a differentially expressed miRNA in this comparison. The reported approximate p value refers to a one-sided, two-sample Kolmogorov-Smirnov testing for the equality between dme-miR-14-3p predicted target and non-target distributions. The number of observations for the predicted target and non-target distributions are identical, as a result of a sampling procedure implemented on both distributions to normalise for 3’UTR length, a potential confounding factor in this type of analysis. Further details of the sampling method can be found in the reported methods section of this study. Comparison: Mated female abdomen vs. virgin female abdomen. This analysis demonstrates that differentially expressed miRNAs in this study do not effect globally significant changes on their predicted targets, implying that target gene regulation may occur specifically on only a subset of predicted miRNA targets.

Remove adaptors

```
#!/usr/bin/perl
use strict;
use Bio::SeqIO;
use lib "/usr/local/Bioperl/lib/perl5/site_perl/5.8.3";
print STDERR "Removes adaptors - also removes short seqs (< 16nt)
and low complexity sequences by default\n";
my $input = shift;
my $adaptor = shift;
if (!$input){
print STDERR "No input file specified\n";
exit();
}
if (!$adaptor){
print STDERR "No adaptor sequence specified\n";
exit();
}

my %seqs;
#print STDERR "Please enter adaptor sequence to match:\n";
#my $adaptor = <STDIN>;
chomp $adaptor;
my $total_count = 0;
my $sequences = Bio::SeqIO->new(-format => 'fasta', -file =>
"$input");
while (my $seq = $sequences->next_seq ) {
$total_count++;
my $sequence = $seq->seq();
my $id = $seq->id();
        if ($sequence =~m/^(\w+)($adaptor\w*)$/){
        my $processed_seq = $1;
        my $adaptor = $2;
        my %bases;
                if (length $processed_seq >= 16){
                        foreach (split('',$processed_seq)) {
                        $bases{$_} = 1 ;
                        }
                        my $bcount = keys %bases;
                        if ($bcount <3) { # low complexity sequence
                        next;
                        }
                        else{
                        $seqs{$processed_seq}++;
                        }
                }
        }
}
foreach my $element (keys %seqs){
my $count = $seqs{$element};
print ">$element\($count\)\n$element\n";}
```

miRNA counts from PatMan

```
#!/usr/bin/perl -w
use strict;
my $input = shift;
my %features;

if (!$input){
print STDERR "Sums feature counts from patman output\n";
print STDERR "$0: <patman file>\n";
exit();
}

open (IN, $input) or die "Can't open file $input\n";
while (<IN>){
      if ($_=~m/^(\S+).*\t+(\S+)\((\d+)\).+/){
      my $feature = $1;
      my $sequence = $2;
      my $count = $3;
      $features{$feature}+=$count;
      }
}
foreach my $element (keys %features){
my $count = $features{$element};
print "$element\t$count\n";
}
```
